# Macondo crude oil from the Deepwater Horizon oil spill disrupts specific developmental processes during zebrafish embryogenesis

**DOI:** 10.1186/1741-7007-10-40

**Published:** 2012-05-04

**Authors:** T Yvanka de Soysa, Allison Ulrich, Timo Friedrich, Danielle Pite, Shannon L Compton, Deborah Ok, Rebecca L Bernardos, Gerald B Downes, Shizuka Hsieh, Rachael Stein, M Caterina Lagdameo, Katherine Halvorsen, Lydia-Rose Kesich, Michael JF Barresi

**Affiliations:** 1 Biological Sciences, Smith College, Northampton, MA 01063, USA; 2 Molecular and Cellular Biology, University of Massachusetts, Amherst MA, USA

**Keywords:** Deepwater Horizon, crude oil, zebrafish, embryonic development, cardiovascular, cartilage, neural crest, peripheral nervous system, somitogenesis, muscle

## Abstract

**Background:**

The Deepwater Horizon disaster was the largest marine oil spill in history, and total vertical exposure of oil to the water column suggests it could impact an enormous diversity of ecosystems. The most vulnerable organisms are those encountering these pollutants during their early life stages. Water-soluble components of crude oil and specific polycyclic aromatic hydrocarbons have been shown to cause defects in cardiovascular and craniofacial development in a variety of teleost species, but the developmental origins of these defects have yet to be determined. We have adopted zebrafish, *Danio rerio*, as a model to test whether water accumulated fractions (WAF) of the Deepwater Horizon oil could impact specific embryonic developmental processes. While not a native species to the Gulf waters, the developmental biology of zebrafish has been well characterized and makes it a powerful model system to reveal the cellular and molecular mechanisms behind Macondo crude toxicity.

**Results:**

WAF of Macondo crude oil sampled during the oil spill was used to treat zebrafish throughout embryonic and larval development. Our results indicate that the Macondo crude oil causes a variety of significant defects in zebrafish embryogenesis, but these defects have specific developmental origins. WAF treatments caused defects in craniofacial development and circulatory function similar to previous reports, but we extend these results to show they are likely derived from an earlier defect in neural crest cell development. Moreover, we demonstrate that exposure to WAFs causes a variety of novel deformations in specific developmental processes, including programmed cell death, locomotor behavior, sensory and motor axon pathfinding, somitogenesis and muscle patterning. Interestingly, the severity of cell death and muscle phenotypes decreased over several months of repeated analysis, which was correlated with a rapid drop-off in the aromatic and alkane hydrocarbon components of the oil.

**Conclusions:**

Whether these teratogenic effects are unique to the oil from the Deepwater Horizon oil spill or generalizable for most crude oil types remains to be determined. This work establishes a model for further investigation into the molecular mechanisms behind crude oil mediated deformations. In addition, due to the high conservation of genetic and cellular processes between zebrafish and other vertebrates, our work also provides a platform for more focused assessment of the impact that the Deepwater Horizon oil spill has had on the early life stages of native fish species in the Gulf of Mexico and the Atlantic Ocean.

## Introduction

More than 200 million gallons of crude oil were released from the Macondo Well in the Gulf of Mexico during the 2010 Deepwater Horizon oil spill [1,2]. The oil gushed from approximately 5,000 feet below the surface creating underwater plumes of oil and slicks on both the ocean floor and surface. The oil's total vertical exposure to the water column suggested it had the potential to impact a diversity of ecosystems in the Gulf of Mexico and Atlantic Ocean from coastal wetlands to coral reefs to deep-water benthic communities [3,4]. In fact, many thousands of invertebrate and vertebrate carcasses have been discovered and attributed to the Macondo oil, and due to the wide-spread and hidden nature of this disaster, species mortality rates are hypothesized to be at least 50 times underrepresented [4,5].

Of particular concern is how embryos and larvae will be affected by this oil spill, as organisms in these early life stages are typically unable to flee a contaminated area and lack the tissue mass and mature detoxification systems necessary to withstand toxic insults. Furthermore, loss of whole clutches of embryos and larvae can have catastrophic consequences for population dynamics over long time periods [6]. The National Oceanic and Atmospheric Administration (NOAA) reported an unusually high number of stillborn and newborn dolphins that began washing up on the Gulf shores starting in January 2011 [7]. While only a small number of these dead dolphins had obvious signs of oil exposure, the timing suggests an association between the gestational period of dolphin embryonic and fetal development with the Deepwater Horizon blowout and oil spill [4]. There are 52 taxa of native fish species also known to spawn in the Gulf of Mexico such as tuna, red snapper and yellowfin groupers [8], and unfortunately little is known about how the Macondo crude oil might directly impact early development of dolphins or these fish species. It will be critically important to identify the molecular targets of crude oil toxicants that effect embryonic development to provide greater predictive power in the risk assessment of this and future spills.

Exposure to crude oil and/or the water-soluble, polycyclic aromatic hydrocarbons (PAH) that make up much of the oil have been shown to cause cardiovascular abnormalities and pericardial edema in topsmelt and Pacific herring embryos, as well as additional defects in jaw and spinal cord development in the crimson-spotted rainbow fish [9-11]. Whether oil from the Macondo well can cause similar defects in embryonic development needs to be determined. The majority of research using native Gulf fish species to determine the effects of crude oil on embryonic development has been limited to the analysis of mortality and gross morphological changes. However, the relatively recent characterization of zebrafish (*Danio rerio*) as a laboratory fish model system has revealed significant insights into the developmental mechanisms governing embryogenesis [12,13]. Zebrafish provide researchers with the consistent accessibility of genetically defined lines of embryos and a battery of reliable genetic, molecular and cellular techniques for higher resolution analysis, benefits that are providing significant advantages to the use of zebrafish in toxicology. The small adult fish size, large embryo clutches, *ex utero *development, and transparent embryo and larval stages of zebrafish enable cost effective maintenance of many fish, reproducible sample sizes, simple application of toxin treatments, and easy evaluation of end-point toxicity [14-18]. The use of zebrafish to assay drug and pollutant toxicity has already provided insights into the developmental and molecular mechanistic roles of metals [19,20], dioxins [21-24], pesticides [25,26], endocrine disruptors [27,28], alcohols [29-31], chemotherapies [32-34] and specific pharmaceutical compounds many of which were assessed via high-throughput screening [35].

Examination of the effect of crude oil and its more common PAHs on zebrafish embryos has shown developmental abnormalities that include cardiac edema, reduced jaw development, curvature of the spine, hemorrhaging, reduced larval heart rate and cardiac arrhythmia [6,36-39]. While these varied deformations are clearly attributed to crude oil exposure, it is unknown what early embryonic processes at the cellular or molecular levels are directly affected. Further investigations utilizing zebrafish to elucidate the molecular mechanisms that interact with and mediate crude oil effects could guide future preventative measures to oil spills and their clean up procedures, as well as yield new strategies to combat the teratogenesis associated with oil spill exposure.

In the present study, we sought to determine whether water-soluble components of the crude oil from the Deepwater Horizon oil spill could disrupt normal embryonic and larval development in zebrafish. Embryos were exposed to water accumulated fractions (WAFs) made from crude oil sampled from the riser insertion tube attached to the Macondo well during the 2010 oil spill. Using an array of high resolution assays to characterize a variety of discrete developmental processes, we show that the Macondo oil does not cause general and wide-spread toxicity but rather disrupts specific developmental processes, some of which have never been reported previously for any crude oil source. We show that WAF-induced deformations in cardiovascular and craniofacial development, observed in this and likely other studies [6,36-39], are associated with reductions in specific rostral migratory streams of cranial neural crest cells. Surprisingly, Macondo oil exposure also generated novel phenotypes in apoptosis, sensory and motor axon pathfinding, somitogenesis and muscle fiber type development, which provide causative evidence for the locomotor swimming behaviors exhibited by WAF-treated embryos. Lastly, we demonstrate that dose and timing of WAF exposure are equally important for the induction of cell death and muscle specific phenotypes. Our results suggest that crude oil components may interact with important molecular mechanisms to influence embryogenesis. The severe teratogenic effects in zebrafish associated with the Deepwater Horizon oil are cause for concern if native fish species in the Gulf of Mexico responded similarly. Finally, our findings provide a more comprehensive phenotypic map to assess pollution effects on development.

## Results

### Gross morphological deformations

Water accumulated fractions (WAF) of the Macondo crude oil were vortex mixed in embryo medium at a 1:10 dilution in accordance with conventional methods [40]. Analysis of different mixing procedures and serial dilution experiments were performed and are described in Additional files 1 and 2. Based on our dose response analysis we conducted all embryo treatments at the 100% concentration of the vortex-mixed, 1:10 WAF stock solution, which will from here on be referred to as WAF. Only during our analysis of locomotor behavior was 50% WAF solution used. These WAF preparations serve to model the portions of the Macondo crude oil sampled from the riser insertion tube during the Deepwater Horizon oil spill that are capable of dissolving in embryo medium. It is important to acknowledge that according to communications with BP during the period of 2 and 3 May 2010 when this sample was being collected Nalco EC9323A defoamer was being applied topside and methanol with VX9831 oxygen scavenger/catalysts solution injected subsea. While we cannot rule out the possibility that these additional agents in the vicinity of the spill site could be present in this sample, it is highly unlikely due to the method of direct sampling through the riser tube.

**Figure 1:**
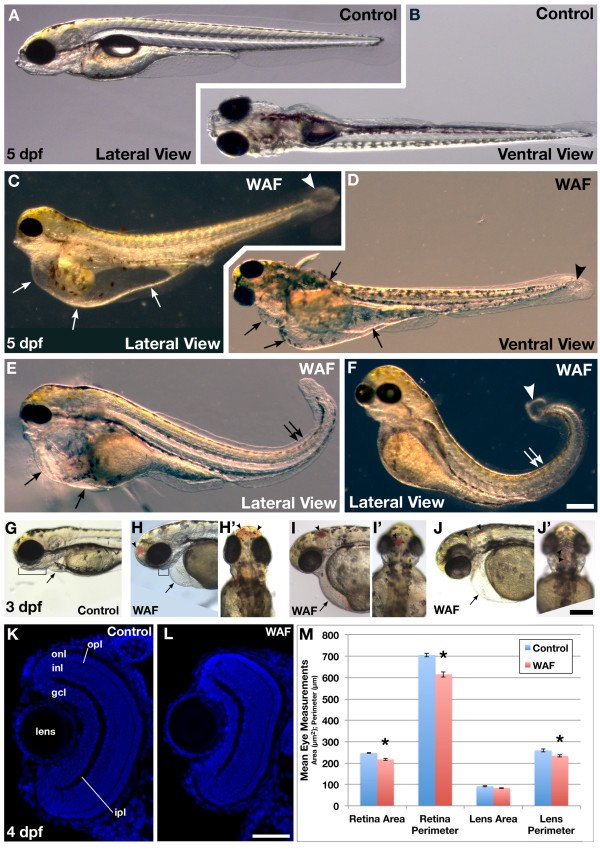
**Exposure to Macondo crude oil-derived WAFs induced diverse gross morphological deformations in zebrafish embryos**. (**A-F**) Lateral and ventral views of live untreated control (A, B) and WAF-treated embryos (C-F) at 5 dpf. Severe cardiac and yolk edema (C, D, E, arrows), dorsal tail curvature (E, F, double arrows), and cysts at the tip of the tail (C, D, F, arrowhead) were visible. (**G, H**) WAF-treated embryos (H) had reduced jaws compared to controls (G, brackets). (G-**J**) At 3 dpf cardiac edema was evident in WAF-treated embryos (arrows), and 28% of embryos had hemorrhaging in the forebrain, midbrain and hindbrain (arrowheads). Lateral (G, H, I, J) and dorsal views (H', I', J'). (**K-M**) Retinal architecture appeared normal in control and WAF-treated embryos (K, L) but there was a slight reduction in the area and perimeter of WAF-treated retinas (M). Except for lens area, the size reductions were statistically significant (M, asterisks; *t*-tests: lens area, *P *= 0.015; lens perimeter, *P *= 0.007; retina area *P *< 0.0005; retina perimeter, *P *< 0.0005). Scale bars: 200 μm, F, J'; 50 μm, L. Abbreviations: gcl, ganglion cell layer; ipl, inner plexiform layer; inl, inner nuclear layer; opl, outer plexiform layer; onl. outer nuclear layer.

Embryos exposed to WAF starting at 3.5 hours post-fertilization (hpf) until a maximum of 5 days post-fertilization (dpf) caused an array of morphological phenotypes that included dorsal tail curvature, cardiac edema, cyst formation, reduced head structures and brain hemorrhages (Figure 1). More specifically, WAF-treated embryos showed moderate (Figure 1F) to severe cardiac edema (Figure 1C-E, arrows) that, in some cases, also included yolk sac edema (Figure 1C, right most arrow). In addition, a dorsal curling of the tail and caudal cyst formation were variably observed within a treated clutch of embryos but were consistently present across treatments (Figure 1E, F, double arrows; Figure 1C, D, F, arrowhead). Qualitatively, the overall size of the brain and eyes were often reduced, which was most apparent in the ventral jaw structures (Figure 1G, H, brackets). Equally obvious was the average 28% of embryos with brain hemorrhages present in the forebrain, midbrain, or hindbrain regions (Control 0.0%, n = 452; WAF, n = 441; Figure 1H-J, arrowheads). While the eyes of some WAF-treated embryos were noticeably smaller, the mean measurements of the area and perimeter of the retina and lens showed only subtle, but statistically significant, reductions in size (Figure 1K-M). These varied, but relatively specific, morphological deformations suggest that certain embryonic processes may be affected by exposure to Macondo crude oil.

### Chemical analysis of water accumulated fraction

**Figure 2:**
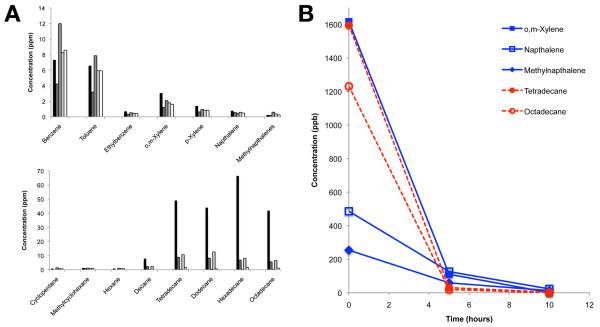
**Compound composition of Macondo crude oil-derived WAFs**. **A**) Concentrations of selected components in samples taken from four different WAF experiments. The most prominent components were aromatics and alkanes. See Supplementary Information for details. **B**) Selected aromatic (blue) and alkane (red) WAF components decreased in concentration over a 10-hour test period.

To determine what components of the crude oil were released into the WAF mixture and thus exposed to the embryos during experimental treatments, WAF samples were analyzed within one hour of being made using solid phase microextraction (SPME, 100 mm polydimethylsiloxane) and gas chromatography mass spectrometry (GCMS, Agilent 7890A GC/5975C MSD). Chemical analysis confirmed the presence of *n*-hexane, toluene, xylene, benzene, naphthalene and ethylbenzene that were reported by BP to be present in this source oil B. This analysis also revealed a variety of additional components, of which the most prominent were aromatics and alkanes (Figure 2A), as observed in chemical analyses of Gulf of Mexico waters after the Deepwater Horizon blowout [41-43]. Aromatic concentrations were similar over the four WAF solutions sampled (Figure 2A). We interpret the large variation in heavier alkane concentrations (Figure 2A) to the inclusion of a non-aqueous phase, into which these poorly soluble components fractionate. Such partitioning of these less-soluble components into deep-plume oil droplets [42] and into the surface slick [43] was observed in the Gulf of Mexico after the BP Oil Spill. For a full detailed report of the chemical analysis obtained see Additional file 2.

To determine how component concentrations changed with time, samples from a WAF solution were taken every five hours from petri dishes maintained under the same conditions as the treated zebrafish embryos. As demonstrated by a few selected representative components, there is a dramatic drop-off in concentrations with time (Figure 2B). For aromatics (blue), the drop-off is less extreme for the heavier napthalenes (75 to 80% drop in five hours), compared to the lighter alkylated benzenes (93 to 95% drop in five hours). The initial concentrations for the heavy alkanes (red) are beyond reported hydrocarbon solubilities, such that the WAF solution is likely to have been initially fully saturated with these poorly soluble components (as discussed further in Additional files 1 and 2). Assuming initial saturation, the drop-off for tetradecane is similar to the heavier napthalenes, at 75% within five hours. These results suggest that while embryos are initially exposed to high levels of hydrocarbon compounds, within the 5- to 10-hour window, they are experiencing hydrocarbon concentrations that approach levels observed in underwater plumes in the Gulf of Mexico [41,42] and those along the Louisiana marshes capable of altering gene expression in adult killifish [44]. For example, within 10 hours, our concentrations of toluene, ethylbenzene and total xylenes fall below ranges observed in underwater plumes [42].

### Cell proliferation and cell death

We next wanted to characterize the gross morphological phenotypes we observed at higher resolution to glean better insight into whether exposure to Macondo oil affected specific developmental processes during zebrafish embryogenesis. WAF-treated embryos exhibit subtle reductions in brain and eye size and changes to the symmetric elongation of the tail (Figure 1), all of which could indicate reductions in cell numbers. No obvious greying of tissue, indicative of cell necrosis, was ever observed in WAF treated embryos; reduction in tissue size could be attributed to either reduced cell proliferation or increased programmed cell death.

**Figure 3:**
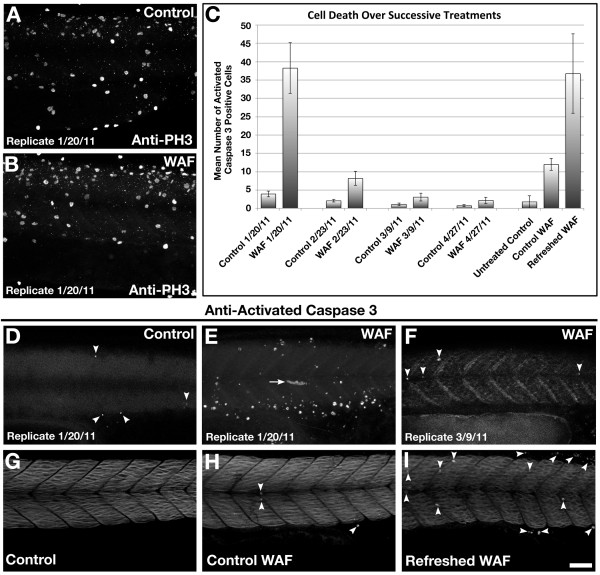
**Macondo crude oil exposure did not affect cell proliferation but did induce programmed cell death**. (**A**, **B**) Phospho-Histone H3 labeling of cells in mitosis were unaffected in 30 hpf WAF-treated embryos (B). (**C**) Quantification of anti-Activated Caspase 3-positive cells in 30 hpf control and WAF-treated embryos over 4 replicates and a WAF-refreshing procedure. The number of apoptotic cells decreased with each successive replicate, but increased following application of freshly-mixed WAF. (**D-F**) Activated caspase-3 labeled 30 hpf control (D, arrowheads) and WAF-treated embryos (E) from experiments in January 2011, and a WAF-treated embryo from an experiment in March 2011 (F, arrowheads). There was a significant decrease in the number of apoptotic cells in WAF-treated embryos between January (E) and March (F). (**G-I**) Representative images from the refreshed WAF experiments (arrowheads denote positive anti-Caspase 3 cells). Embryos were either untreated (G), exposed to the same WAF from 3.5 hpf to 30 hpf (H), or exposed to WAF from 3.5 hpf to 15 hpf and then exposed to a fresh WAF solution from 15 hpf to 30 hpf (I). Embryos in the refreshed WAF group (I) partially recovered the cell death phenotype of earlier replicates (E). (A, B, D-I) Lateral trunk views centered on somites 14 to 21. Scale bar 50 μm, A, B, D-I.

Embryos treated with WAF from 3.5 hpf to 30 hpf were immunolabeled for Phosphorylated Histone H3 (PH3) to visualize all cells undergoing mitosis [45]. Pooled averaging of three replicate WAF treatments did not show significant changes in the number of PH3-positive cells as compared to untreated controls (Figure 3A, B; control, 79.9; WAF, 76.2; *t*-test, *P *= 0.18). To control for the presence of outlying values, the lack of statistical significance was confirmed with a non-parametric test (Mann-Whitney, *P *= 0.0511). If mitotic rates are not altered in WAF-treated embryos, then reductions in tissue size may be due to increased apoptosis or programmed cell death.

To test whether cells were dying by apoptosis we immunolabeled embryos for Activated Caspase 3, which is a marker for cells undergoing programmed cell death [46]. Pooled averaging of four replicate experiments did show a statistically significant increase in the number of dying cells positive for Activated Caspase 3 along the trunk in WAF-treated embryos as compared to controls (Figure 3C; control, 1.94; WAF, 12.9; *t*-test, *P *= 0.0001). Apoptotic cells were present both inside and outside the spinal cord (Figure 3D-F). Interestingly, despite using the same crude oil source for each WAF preparation, the number of apoptotic cells in WAF-treated embryos decreased with each successive replicate experiment (Figure 3C). In the first treatment an average of 38.25 Activated Caspase 3-positive cells were seen in WAF-treated embryos (Control, 3.9; *t*-test *P *= 0.0001), and in the second treatment this average dropped to eight cells (Control, 2.05; *t*-test *P *= 0.0062). Furthermore, in the third and fourth replicate experiments the number of apoptotic cells in WAF-treated embryos was no longer statistically different from their respective controls (Rep3: control, 1.1; WAF, 3.05 *t*-test *P *= 0.0966; Rep4: control, 0.7; WAF, 2.15; *t*-test *P *= 0.1151). A total of 20 embryos were examined for each condition in each separate replicate experiment.

The prevalence of WAF-induced cell death during the initial experiments suggests our crude oil sample was changing and becoming less potent over time. Chemical analysis did show much of the aromatics and alkanes in the WAF drop off dramatically within five hours after exposure to the embryos (Figure 2B), thus it is possible that our crude oil source sample experienced an incremental loss of some compounds over the course of storage. To test this hypothesis directly, we treated embryos with WAF from 3.5 hpf to 18.5 hpf and then replaced this solution with a freshly mixed WAF solution and continued the experiment until 30 hpf. This WAF refreshing protocol did show a statistically significant increase in the number of Activated Caspase 3 cells (36.74 cells; n = 42) as compared to untreated (1.75 cells; n = 40) or non-refreshed WAF treated controls (11.97 cells; n = 38) (*Kruskal-Wallis Test P *< 0.0005) (Figure 3G-I). In fact, the number of apoptotic cells in the refreshed WAF experiment was similar to the initial WAF experiment (Rep1, 38.25 cells; *P *= 0.2276) (Figure 3C). This result suggests refreshing the WAF solution returned the cell death-inducing properties of the crude oil.

### Circulation and vasculogenesis

**Figure 4:**
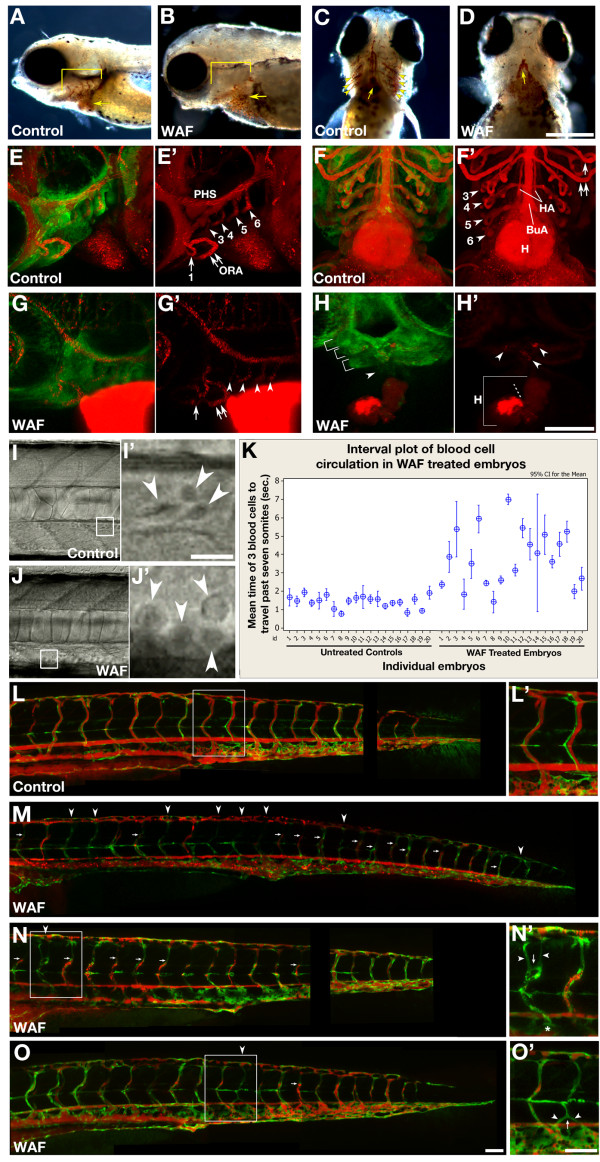
**Defects in head and trunk vascular development result in reduced circulatory function**. (**A-D**) Hemoglobin staining revealed a reduction in the amount of blood cells in 3 dpf WAF-treated embryos, notably in the vasculature of the pharyngeal arches (A, B, brackets, lateral view; C, D, arrowheads, ventral view), with staining abruptly ending in the heart or bulbus artery prior to filling the aortic arches (arrow). (**E-H'**) Microangiography analysis with QTracker 655 fluorescent quantum dots (red) injected into 3 dpf *tg[fli:eGfp] *transgenic larvae to visualize endothelial cells associated with the vasculature (green). Endothelial vasculature in moderately affected WAF-treated embryos (G) was comparable to controls (E), however in severe cases posterior arch vasculature was lost and circulation was reduced (H, brackets, arrowhead, H', arrowheads). (E', F', G', H') Arrowheads and arrows denote the specific blood vessels associated with the pharyngeal arches. Accumulation of quantum dots in the heart atrium suggests reduced flow into the ventricle (H', dashed line). (**I-K**) Real time analysis of the flow speed of individual blood cells (I-J', arrowheads) over a 7-somite distance in the dorsal aorta. WAF-treated embryos have reduced blood circulation (K, right half of graph). (**L-O'**) Intersegmental blood vessels had reduced circulation of quantum dots as demonstrated by either a complete absence of flow (M-O, arrowheads) or truncated flow (M-O arrows). Ectopic branching and vascular remodeling was evident in some segments devoid of circulation (N', O'). Abbreviations: BuA, bulbus arteriosus; H, heart; HA, hypobranchial artery; ORA, opercular artery; PHS, primary head sinus. Numbers and affiliated arrowheads in E' and F' represent the first through sixth aortic arch. Scale bars = 200 μm, A-D; 100 μm, E-H'; 50 μm, I, J, N', O'; 20 μm, I', J'; 50 μm, L-O.

We have demonstrated that embryos treated with WAF derived from the Macondo Oil exhibit cardiac edema and hemorrhaging in the brain (Figure 1). We next wanted to determine whether the Macondo Oil WAF likewise interferes with the proper development and physiology of the circulatory system that has been demonstrated previously for other crude oil types and components [9,10,36-39,47,48]. Closer examination for the presence of blood through whole-mount hemoglobin stained control and WAF-treated embryos showed a qualitative reduction in the amount of blood cells in treated embryos (Figure 4A-D). This reduction was particularly obvious in the vasculature of the pharyngeal arches (Figure 4A, B, brackets; C, arrowheads, D). Importantly, significant hemoglobin staining was still present in the common cardinal vein distal to and including the heart, but abruptly ended in the heart or bulbus artery prior to filling the aortic arches (Figure 4A-D, arrow).

The reduced blood volume specifically beyond the heart could be due to diminished hematopoiesis, improper vessel formation or altered heart function. To better discern between these possibilities, we conducted angiography with fluorescent quantum dots in 72 hpf live embryos treated with WAF starting at 3.5 hpf. This procedure was done in *tg[fli:eGfp] *transgenic larvae, which allowed for visualization of green endothelial cells as the red quantum dots flowed throughout the circulatory system, illuminating both the extent of circulatory function and blood vessel anatomy [49]. Imaging of the aortic arches revealed that in some cases the endothelial vasculature was present and capable of supporting circulation (Figure 4E, G, arrows and arrowheads), while in more severe cases the more posterior arch vasculature was lost and circulation was significantly reduced in the remaining vasculature (Figure 4F, H). In addition, there was often a build-up of the quantum dots in the heart atrium paired with a reduced flow of these quantum dots into the ventricle (H', dashed line). These results suggest that the endothelial vasculature and proper heart development and function are all variably compromised in WAF-treated embryos. Further supporting these results, real time imaging of blood flow through the dorsal aorta showed that individual blood cells from 60 different WAF-treated embryos (3 replicates) took, on average, 2.68 times longer to travel the distance of 7 somites (Figure 4I-K; Control, 1.44s; WAF, 3.85s; *t*-test, *P *= 0.0001).

Continued analysis of quantum dot circulation and endothelial vasculature in the trunk revealed additional phenotypes involving intersegmental blood vessel development. The most prevalent phenotype was varied frequency of reduced circulation of fluorescent quantum dots through the intersegmental blood vessels, such that vessels showed either a complete absence of flow (Figure 4L-O, arrowheads), truncated flow (Figure 4L-O, arrows), or normal circulation. Interestingly some vessels devoid of any quantum dots often showed ectopic branching and an overall improper vessel pattern (Figure 4N', O'). These phenotypes were consistent over three separate experimental replicates with at least 10 embryos imaged per replicate. As a whole, these results suggest the Macondo crude oil is capable of causing specific deformations in vasculogenesis in both the trunk and head of zebrafish that leads to reduced circulatory function.

### Craniofacial development

**Figure 5:**
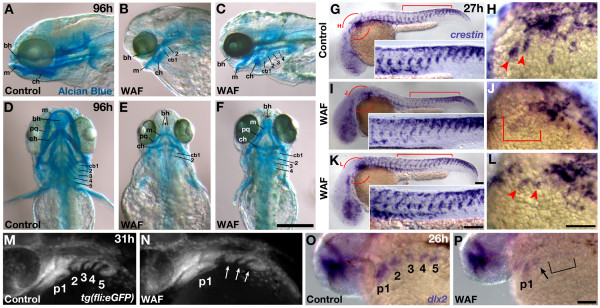
**Craniofacial defects induced by Macondo crude oil exposure were correlated with defects in neural crest development**. (**A-F**) Alcian blue staining of head and jaw cartilage in 4 dpf control (A, D) and severely (B, E) or moderately (C, F) affected WAF-treated embryos. WAF-treated embryos had a variable reduction in the size of all cartilage components, notably a lack of anterior extension of jaw elements and a dramatic reduction in posterior pharyngeal arches (B, C, E, F). (**G-L**) Whole mount *in situ *hybridization of *crestin *expression in neural crest cells. *crestin *expression is normal in the trunks of control and WAF-treated embryos (G, I, K, bracket; circles in G, I, K represent magnified view in H, J, L). However, *crestin *expression was variably reduced specifically in the anterior migratory streams, an area of cells that will populate the pharyngeal arches (H, J, L, arrowheads and bracket). (**M, N**) Cranial neural crest forming pharyngeal arches (p1-5) at 31 hpf as visualized by *fli *driven expression of GFP. One of the posterior-most pharyngeal arches is missing in WAF treated embryos (N, arrows) as compared to controls (M, 3, 4, 5). (**O, P**) *dlx2 *expression in the region of pharyngeal arches is reduced in 26 hpf WAF-treated embryos (P, p1, arrow, bracket) as compared to controls (O). *dlx2 *expression is nearly lost in the most posterior regions of the presumptive pharyngeal arches (P, bracket) despite robust expression still seen in the forebrain. Abbreviations: bh, basihyal cartilage; cb1-5, ceratobranchial branches; ch, ceratohyal; m, Meckel's; pq, palatoquadrate. Scale bars = 200 μm, A-F; 100 μm, G-L.

Due to the vascular defects associated with pharyngeal arches, we next examined whether exposure to Macondo crude oil similarly causes defects in craniofacial development, a phenotype known to occur with specific PAH exposure [11,36]. We treated embryos from 3.5 hpf to 4 dpf and then performed Alcian Blue staining to visualize cartilage [50]. Consistent with head vasculature phenotypes, WAF-treated larvae showed a range of head and jaw cartilage phenotypes (Figure 5A-F). WAF-treated embryos had a general size reduction in all cartilage elements with a significant lack of anterior extension of the most prominent jaw elements (Meckel's and ceratohyal cartilage) (Figure 5B, C, E, F). The most severely affected embryos showed dramatic reduction in all of the pharyngeal arch cartilage elements (ceratobranchial) with a complete loss of the most posterior three arches as compared to untreated control larvae (Figure 5A, B, E). In addition, we report here for the first time, in response to any crude oil treatment, a lack of anterior midline fusion of the basihyal cartilage (Figure 5E, arrowhead). Less severely affected embryos, however, did not display this basihyal phenotype (Figure 5F).

Because of the corresponding deformations in both endothelial and cartilage cell development in the head, we next hypothesized that these phenotypes may be derived from an earlier defect in the differentiation or migration of neural crest cells, which are a common progenitor cell contributing to pharyngeal arch cartilage, smooth muscle of the pharyngeal arch arteries as well as portions of the heart such as the arterial pole and endocardial cushions [51-56]. To test this we conducted whole-mount *in situ *hybridizations for *crestin *transcripts in WAF-treated and control embryos at 27 hpf. *crestin *is a known marker of neural crest cells during their early specification, delamination from the dorsal neural tube, and subsequent migration into the characterized pathways of the trunk and head [57]. Interestingly, embryos treated with WAF from 3.5 hpf to 27 hpf of development showed qualitative reductions in *crestin-*labeled cells, specifically in the anterior migratory streams known to populate the pharyngeal arches (Figure 5G-L; H, J, L, arrowheads and bracket). In contrast, the amount and position of *crestin-*positive cells in the trunk qualitatively appeared normal (Figure 5G, I, K, insets).

To confirm that deformations in pharyngeal arch development were associated with defects in early cranial neural crest populations, we examined the presence of rostral migratory streams of neural crest cells in *tg(fli:GFP) *transgenic embryos and whether they appropriately expressed the cell specification marker *dlx2 *[58,59]. A total of 76% of WAF treated embryos (n = 83) showed a specific loss of one of the more posterior pharyngeal arches (Figure 5M, N, arrows) (control = 8.6%; n = 83; *P *< 0.0005). Importantly, despite normal *dlx2 *expression in the forebrain, 89.4% of WAF-treated embryos (n = 94) showed a significant reduction of *dlx2 *expression in all of the rostral migratory streams with a near complete absence in the presumptive posterior-most arch locations (Figure 5O, P, bracket) (control = 15.6%; n = 122; *P *< 0.0005). These findings suggest that Macondo crude oil is compromising the early development of cranial neural crest cell differentiation, which results in reduced posterior pharyngeal arches and the more visible defects in the heart, arch vasculature and craniofacial development.

### Locomotor behavior

**Figure 6:**
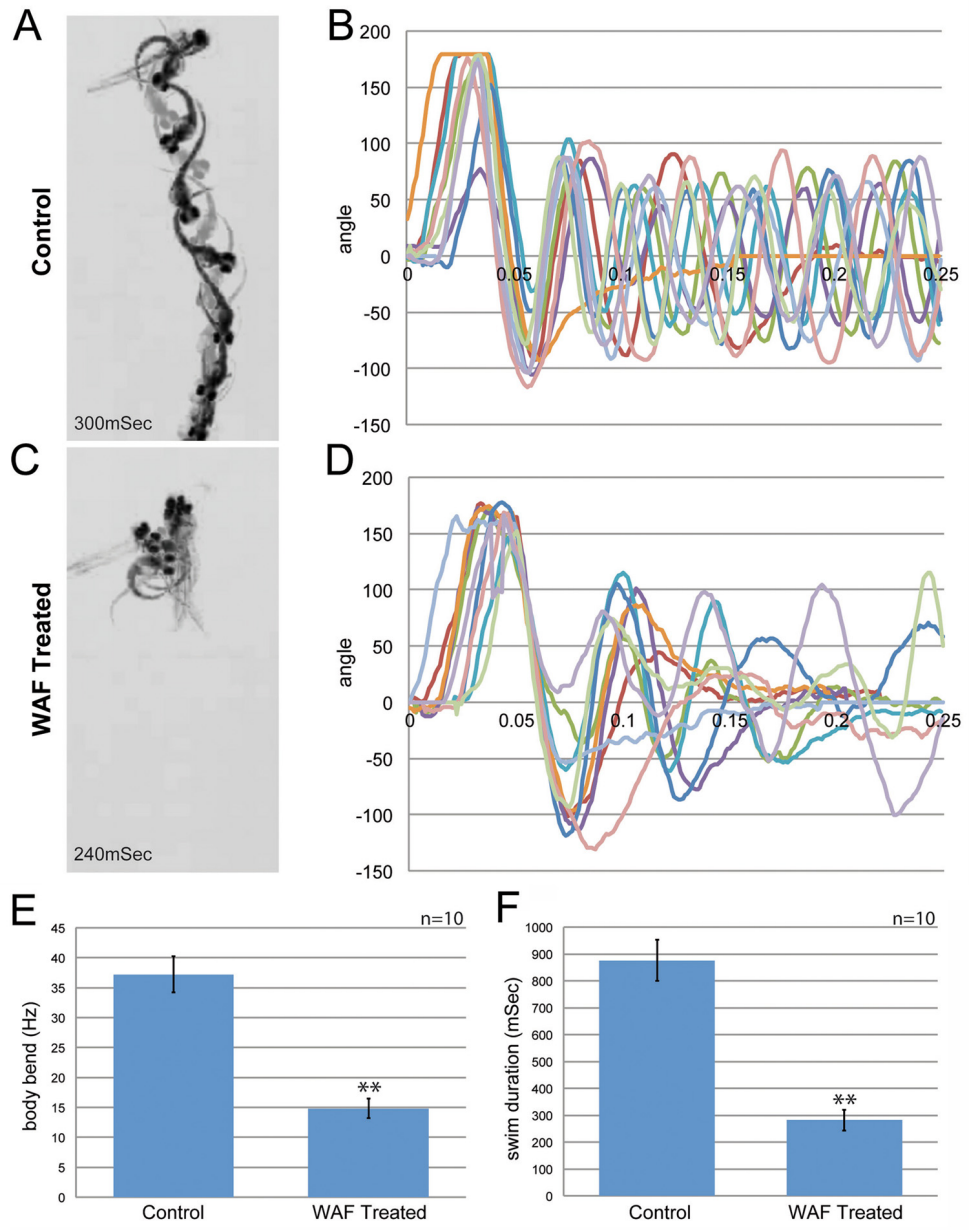
**Macondo crude Oil exposure impaired escape behavior by 48 hpf**. (**A**) Individual frames from high-speed video recordings are shown for control larvae. The images are overlaid in 20 mS intervals and the duration of the response captured within the field is indicated. (**B**) Kinematics traces are shown for 10 escape responses each for control larvae. 0° indicates a strait body and positive and negative angles represent body bends in opposite directions. The time is indicated in seconds. (**C**) Image overlays for a WAF-treated larva escape response illustrates the failure to clear the field that was frequently observed. (**D**) Kinematic traces for WAF-treated larvae reveal reduced, abnormal body bend frequencies. (**E**) Quantification of body bend frequencies. (**F**) Quantification of the duration of escape responses reveals that WAF-treated larvae respond for shorter periods of time. Asterisks in E and F indicate statistically significant differences (n = 10, *P *< 0.01).

During our many WAF treatments it was clearly evident embryos displayed irregular swimming behaviors. The locomotor escape response to stimuli is an important survival behavior that develops later in embryogenesis. Interestingly, previous studies examining the effect of PAHs on zebrafish swimming behavior did not reveal any significant phenotypes [36]. Therefore, we systematically tested whether exposure to Macondo crude oil WAF impacted swimming patterns and escape responses. To do this, we recorded the swimming behavior of individual 48 hpf larvae with a high-speed video camera (1,000 frames/second) following the administration of a specific touch stimulus [60]. WAF-treated embryos demonstrated abnormal swimming behavior and a failure to escape based on multiple criteria. WAF-treated embryos showed reduced sensitivity to touch stimuli, as demonstrated by 70% response rate for WAF-treated embryos as compared to a 99% response rate for untreated control embryos (n = 100 trials from 10 embryos each). When a response was produced in WAF-treated embryos they showed a significantly reduced frequency of body bends (Control, 39.10Hz; WAF, 18.82Hz; n = 10 each; *t*-test, *P *< 0.01) and swam for less time than untreated control embryos (Control, 875.8mS; WAF, 282mS; n = 10 each; *t*-test, *P *= 0.01) (Figure 6). The presence of locomotor behavior phenotypes suggests that there could either be a problem with neural transmission or a developmental problem resulting from an anatomical deformation in the nervous or musculature systems.

### Neuronal development in the central and peripheral nervous system

Development of locomotor movement in response to touch stimuli requires a complex neural network that begins with an elaborate meshwork of Rohon Beard sensory axons at the periphery that originate from the dorsal neural tube. These bipolar sensory neurons make connections with a variety of interneurons that function to relay signals to Mauthner neurons, which serve as the major motor control center in the hindbrain. Mauthner neurons then send signals back down the spinal cord to stimulate coordinated motor neuron activation to achieve the alternating contraction of skeletal muscle required for swimming behaviors [61-63]. In order to determine what underlying developmental processes may be contributing to the visible defects in locomotor behavior, we used specific antibody markers to visualize each cellular step in the locomotor neural circuit.

**Figure 7:**
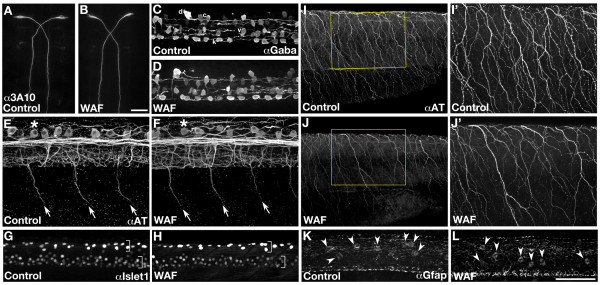
**Macondo crude oil exposure caused specific deformations in the peripheral but not central nervous system**. (**A-L**) 30 hpf control and WAF-treated embryos labeled by immunohistochemistry. (A, B) 3A10 labeled Mauthner neurons in the hindbrain were normal. (C, D) Distribution of Gaba-positive interneurons in the spinal cord were not impacted by WAF treatment (d, DoLa; c, CoSa; v, VeLD; k, KA). (E, F) Anti-Acetylated tubulin (AT) labeling of primary motor axons (arrows) or Rohon Beard sensory neuronal somas (asterisk) were correctly positioned in WAF-treated embryos (lateral view of trunk and spinal cord). (G, H) Qualitatively, Islet1 labeling for primary and secondary motor neurons (lower bracket) and Rohon Beard sensory neurons (upper bracket) were positioned normally. (I, J) The branching pattern of AT-labeled sensory axonal projections along the trunk epidermis was significantly reduced (I', J', magnified views of boxed area in I, J). (K, L) Anti-Gfap labeled radial glia somas in WAF-treated embryo spinal cords (L) were correctly positioned in the ventricular zone (arrowheads) and similar in number to controls (K). (A, B) Dorsal views of the hindbrain. (C-L) Lateral views of the spinal cord (C, D, E, F, G, H, K, L) and trunk (I, J). Scale bars = 50 μm, A-L.

Anti-Acetylated Tubulin (AT) labels all axonal pathways in the zebrafish as well as specifically marks the somas of Rohon Beard sensory neurons [64]. Anti-Islet-1 labels both the nuclei of Rohon Beard Sensory neurons and motorneurons, anti-Gaba labels a series of interneurons, and the 3A10 antibody marks Mauthner neurons in the hindbrain [65-67]. Surprisingly, using this spectrum of neuronal markers we found only one consistent and specific anatomical deformation that was restricted to the peripheral projections of sensory axons. The Mauthner neurons that serve as the central mediators for startle-reflex behavior in the hindbrain, showed absolutely no difference in number or axonal projections between WAF-treated and untreated control embryos (Figure 7A, B; n = 60) [67-69]. Likewise, no statistical difference was seen in the average number or position of Gaba-positive DoLa, CoSA, VeLD or KA interneurons (Figure 7C, D; Control, 41.1 cells, n = 60; WAF, 41.3 cells, n = 59; *t*-test, *P *= 0.855).

Investigation of motorneuron development did not reveal major differences in their cell differentiation. Specifically, anti-AT labeling of primary motor axons did not show consistent defects over the length of the trunk (Figure 7E, F, arrows). It should be noted that while occasional errors in motor axon pathfinding were detected, they were always associated with an affiliated muscle patterning defect that we describe in detail later. Quantification of three separate replicate experiments of Islet1 labeling for primary and secondary motorneurons at 30 hpf showed only minor reductions in the number of motorneurons, with only one of the three replicates actually exhibiting statistical significance (Figure 7G, H, lower bracket; Rep 1: 16% reduction (control n = 11, WAF n = 20), *t*-test, *P *= 0.0001; Rep 2: 4.6% reduction (control n = 18, WAF n = 18), *Mann-Whitney test, P *= 0.456; Rep3: 6.6% reduction (control n = 20, WAF n = 19), *Mann-Whitney test, P *= 0.043). These results suggest that in some limited way motorneuron number may be impacted by exposure to Macondo oil WAF. However, based on the relatively normal pattern of primary motor axons, very modest overall reductions in number, variable significance and time period of analysis correlating with the middle of secondary motorneuron birth [65], we attribute these minor decreases in motorneuron populations more likely to a mild developmental delay in treated embryos rather than any biologically relevant effect from the oil.

Similarly, no qualitative differences were seen in the position or morphology of Rohon Beard sensory neurons as detected with anti-AT labeling (Figure 7E, F asterisks). Quantification of Rohon Beard sensory neuron numbers with anti-Islet1 showed little to no effect (Figure 7G, H, upper bracket). Similar to motorneurons, only one of the three replicates revealed a statistically significant difference (Rep 1: 2% increase (control n = 11, WAF n = 20), *t*-test, *P *= 0.72; Rep 2: 6.3% reduction (control n = 18, WAF n = 18), *t*-test, *P *= 0.184; Rep 3: 15.6% reduction (control n = 20, WAF n = 19), *t*-test, *P *= 0.0001). Again, based on the normal morphology and position of Rohon Beard cells, with no statistical difference present in the first two replicates, and only a small effect observed in the third replicate, we conclude Rohon Beard sensory neuronal number to be unaffected by Macondo oil. However, analysis of the meshwork of sensory axonal projections in the periphery was consistently both visually and quantitatively significantly reduced (Figure 7I, J, Control axonal labeling intensity = 1274.1 pixels, n = 55; WAF axonal labeling intensity = 883.9 pixels, n = 59; *t*-test, *P *= 0.0001). These results suggest most of the anatomical development of the locomotor circuitry is unaffected by Macondo crude oil WAF with the exception of subtle but consistent deformations in the amount and/or branching dynamics of sensory axonal projections.

While the overall number of many key neuronal cell types was normal in WAF-treated embryos, we wanted to confirm that radial glial cells, which serve as the neural stem cell population in the developing spinal cord, were also unaffected [70-72]. Using an antibody to Glial fibrillary acidic protein (Gfap) to mark radial glia we show that radial glial cell staining throughout the spinal cord appeared normal (data not shown). Most relevant was the normal number of Gfap-positive cell somas located at the ventricular surface of the neural tube (Figure 7K, L, arrowheads; Control, 5.99 cells, n = 40; WAF, 5.67 cells, n = 40; *t*-test, *P *= 0.582). Anti-Gfap labeling fills radial glial cell bodies when they are undergoing mitosis at the ventricular zone (Johnson and Barresi, in preparation; [73]). Normal numbers of such dividing radial glia indicates that exposure to Macondo crude oil WAF does not affect neural stem cell proliferation during embryogenesis.

### Somitogenesis and muscle fiber type development

**Figure 8:**
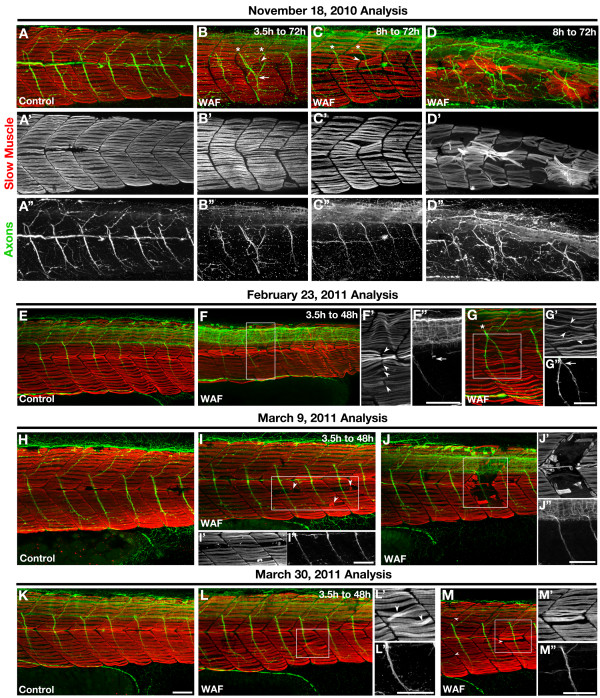
**The severity of deformations in slow-twitch skeletal muscle development decreased with each experimental replicate**. (**A-M**) Lateral views of F59 labeled Myosin heavy chains in slow-twitch muscle fibers (red) and anti-Acetylated tubulin labeled motor axons (green) in the embryonic trunk at 72 hpf (A-D) and 48 hpf (E-M). (A-D) Initial Macondo crude oil WAF treatments beginning at either 3.5 hpf (B) or 8 hpf (C, D) and ending at 72 hpf showed severely defective neuromuscular phenotypes, such as improper somite boundary formation (B, C, arrowhead) or slow muscle loss and disorganization (D). Somite boundary defects associated with the middle or ventral portion of the somite correlated with motor axon pathfinding errors (B, asterisks, arrow; D). (E-M) Subsequent WAF treatments beginning at 3.5 hpf and ending at 48 hpf induced somitogenesis (F, G; F, G', arrowheads), slow muscle (I-M', arrowheads), and motor axon pathfinding (F, G; F", G", arrows) defects, however the severity of these defects decreased over time with each experimental replicate from November to March. (A-M) Primed letters represent single channel images of slow muscle (single prime) or axon (double prime) labeling for the whole image (A-D) or just the boxed regions (F, G, I, J, L, M). Scale bars = 50 μm, A-M".

Because our analysis of the nervous system showed specific deformation restricted to the axonal projections of the peripheral nervous system, it was possible that these defects were secondary to deformations in the paraxial mesodermal environment through which these axonal projections need to navigate. During somite development, radially migrating slow muscle precursor cells provide key guidance cues to the simultaneously outgrowing motor axons [74]. Therefore, any defects in the proper specification, migration and/or position and integrity of slow muscle fibers could influence the development of the peripheral nervous system. We exposed embryos from 3.5 hpf to 48 hpf in Macondo crude oil WAF and assayed for F59 labeling, which preferentially recognizes slow muscle myosin in zebrafish [75,76]. As with most assays, we discovered embryos displaying a range of slow muscle development phenotypes from no observable defects to the loss of muscle fibers and segment boundary errors. Interestingly, similar to what was observed for our cell death analysis over time, embryos displaying slow muscle phenotypes were extremely severe during initial treatments but these phenotypes decreased significantly over the course of several months of repeated experiments using the same crude oil source for WAF preparation (Figure 8).

Initial experiments in November 2010 showed clear defects that represented several distinguishable phenotypes in the treated embryos (Figure 8A-D). The first was the sporadic instance of defects in proper somite boundary formation, such that a segment boundary in a portion of one somite would be absent as represented by slow muscle fibers extending from the anterior boundary of one somite to the posterior boundary of the neighboring somite (Figure 8B, C, arrowhead). Most interestingly, double labeling for motor axons showed pathfinding errors in the affected somite that were directly correlated to the somite/slow muscle defect (Figure 8B, asterisk, arrow). These results suggest that Macondo crude oil may not only be affecting slow muscle fiber development but also somitogenesis, the process by which segment boundaries are established in the paraxial mesoderm [77-79]. The second distinguishable phenotype was the sporadic loss and disorganization of slow muscle fibers in the superficial monolayer (Figure 8D), which suggests impairments in slow muscle differentiation and subsequent migration in WAF-treated embryos.

Later experiments revealed somitogenesis defects with corresponding motor axon pathfinding errors, but these deformations were not as dramatic as compared to our first experiments (Figure 8E-G) and subsequently absent from the final replicates (Figure 8H-M). Later experimentation still showed extremely sporadic loss and disorganization of the slow muscle monolayer (Figure 8H-M). Interestingly, high resolution imaging of regions showing reductions in slow muscle fibers revealed muscle phenotypes resembling muscle degeneration [80-85]. Variable sized fragments of slow muscle myosin were found present in locations devoid of morphologically normal slow-twitch fibers (Figure 8I, J, red). In contrast to the somite boundary defects, motor axon pathfinding appeared normal in areas showing these missing or degenerating muscle fibers (Figure 8I, J, green). Lastly, these phenotypes continued to diminish in severity upon our last round of treatment (Figure 8K-M).

**Figure 9:**
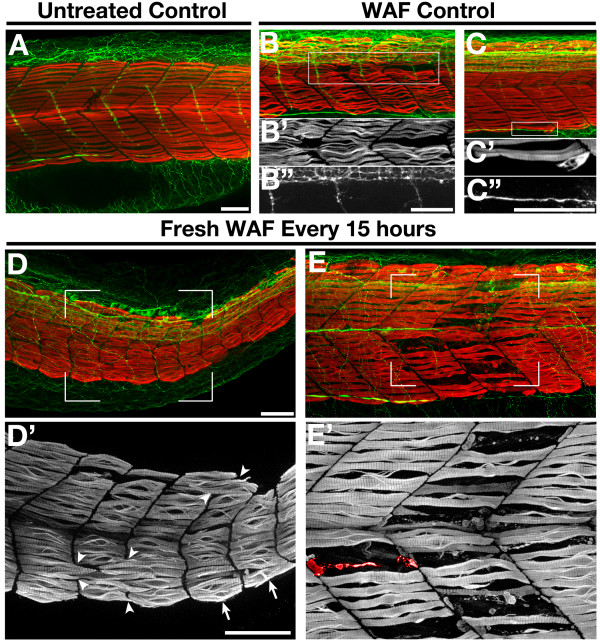
**Repeated application of freshly mixed WAF reproduced the severe skeletal muscle phenotypes**. (**A-E**) Lateral views of the trunk of an untreated control embryo (A), WAF-treated embryos from 3.5 hpf to 48 hpf (B, C), and embryos exposed to freshly mixed WAF every 15 h (D, E). WAF-treated controls showed mild slow muscle defects (B', C'), while embryos treated repeatedly with refreshed WAF displayed severe somite and slow muscle phenotypes (D', E'). As seen in earlier experiments, slow muscle fibers spanned presumptive boundaries (D', arrowheads), somitic shape was irregular (D', arrows), and slow muscle degeneration was evident (E, a representative degenerating myofibril is pseudo-colored red). Scale bars = 50 μm, A-E'.

The sporadic nature of muscle phenotypes both in a clutch of treated embryos and within an affected embryo paired with the gradual loss of these phenotypes over time suggests that the causative agent in the crude oil is not particularly potent and somehow loses its activity over time. Therefore, in an attempt to reproduce the severity of the muscle and somitogenesis defects observed initially, we exposed embryos from 3.5 hpf to 48 hpf in WAF that was refreshed every 15 h. Similar to our results of increased apoptosis in refreshed WAF treatments, more severe muscle deformations were present in refreshed WAF-treated embryos as compared to untreated controls and non-refreshed WAF-treated embryos (Figure 9). The sporadic nature of these deformations was consistent, but the severity was now qualitatively similar to our initial experiments. Remarkably, somitogenesis defects were detected again, showing slow muscle fibers crossing in locations that would normally have a somitic boundary (Figure 9D, D', arrowheads) as well as the presence of irregularly shaped somites (Figure 9D', arrows). In addition, significant slow muscle loss and potential degeneration was also observed following this WAF refreshing protocol. Specifically, slow muscle myosin fibrils were seen in varying stages of degeneration (Figure 9E). This type of dose-response approach confirms that some component(s) within the Macondo crude oil WAF do directly disrupt somitogenesis and slow muscle development.

## Discussion

By all standard measures the Deepwater Horizon blowout caused the worst oil spill the Gulf of Mexico has ever experienced, and recent news reports suggest oil might still be leaking from the Macondo well into the Gulf waters [4,86-89]. To truly assess the total impact of this disaster it will be critical to monitor the fauna and flora of the Gulf of Mexico and Atlantic Ocean for years to come. To make this assessment efficient and thorough, researchers will need to know the range of phenotypes expected from exposure to Macondo oil; however, collecting this information from fish growing within these waters may be particularly challenging. If native species encountered the Macondo oil from the Deepwater Horizon spill during their embryonic or larval stages, these organisms may have died shortly after exposure or were eaten by predators, limiting the number of specimens available for study. Moreover, collection procedures for critical stages of embryonic development and reliable endpoint assays are limited for the analysis of native species from the Gulf of Mexico. In this study we used the tractable zebrafish model system to determine whether water-soluble components of the Macondo oil collected from the riser during the oil spill could directly impact the embryonic development of a bony fish. WAF made from the Macondo oil did not cause wide spread toxicity or even cause significant problems with the earliest developmental processes required by the embryo to progress through cleavage, gastrulation and neurulation. This is surprising, as these early stages of development are arguably the most vulnerable to any environmental teratogen. Rather, our data support a model in which Macondo crude oil WAF cause specific developmental deformations that exhibit both spatial and temporal selectivity.

Many fish species, such as the commercially relevant Atlantic bluefin tuna, red snapper and gag grouper all lay their eggs in the open waters of the Gulf of Mexico near the site of the Deepwater Horizon platform [90-93]. The produced embryos are carried by water currents to the shallows of the Gulf shores to develop into larvae before swimming back into the open ocean as juvenile fry. Unfortunately, the Macondo oil from the Deepwater Horizon disaster was similarly carried along current driven paths to the shores and throughout the Gulf of Mexico [41]. Historically most oil spills have occurred at or near the surface of the water, which would result in limited hydrocarbon dissolution, particularly for more volatile hydrocarbons that are quickly lost to the atmosphere. However, the Deepwater Horizon oil spill is unique in its deep sea origin, which provided the crude oil more time and exposure to the water column creating underwater plumes mostly composed of water-soluble fractions of C_1_-C_3 _hydrocarbons and aromatic compounds [42,43]. Therefore, understanding the impact that water accumulated fractions may have on the development of native species is particularly relevant for this disaster. In fact, our results demonstrate that the hydrocarbon concentrations that produced phenotypes in zebrafish embryos are comparable to the levels detected in the underwater plumes [42,43] and along the Louisiana marshlands that affected gene expression in adult killifish [44]. It is known that interactions between crude oil and various environmental factors, such as temperature, salinity and pressure, will impact hydrocarbon dissolution and PAH uptake [94,95]; however, while our WAFs were largely made in fresh water, the similar ranges of hydrocarbon concentrations recently documented in the Gulf waters following the spill suggest our experiments can provide real insight into the potential risks faced by native species that spawned in the Gulf of Mexico during and after this oil spill.

We observed three broad phenotypes following treatment with Macondo oil WAF. The first observation was a mild but consistent reduction in embryo size paired with changes in head and trunk morphology, and the second phenotype was a compromised cardiovascular system. Both of these broad effects are consistent with responses reported previously to a variety of crude oil sources and specific PAHs [6]. However, we report here for the first time that the Macondo oil WAF does dramatically reduce touch sensitivity and impair proper swimming behavior. Using a variety of molecular and cellular labeling procedures paired with high resolution microscopy we were able to further elucidate the developmental origins behind these three broad observations.

### Defects in size and shape through induction of apoptosis

WAF-treated zebrafish embryos had visible reductions and morphological changes in the size and shape of the head and trunk. These phenotypes could be explained by a reduction in cell proliferation or increase in programmed cell death. We found there was no reduction in the number of mitotic cells (Figure 3A, B), which was further confirmed by no change in the number of dividing neural stem cells (Figure 7K, L). These data suggest cell division rates were unaffected by Macondo crude oil WAF. However, we did detect a statistically significant increase in the amount of apoptosis (Figure 3C-I), supporting a cell death regulatory role for some component in the crude oil. This component could actively induce cell death or play a role in the repression of a survival factor. There are relevant data to support this hypothesis; crude oil, fuel oils, or specific PAHs have been documented to up-regulate known apoptotic proteins in juvenile cod, to increase programmed cell death in cultured dolphin renal cells, and to trigger apoptotic DNA fragmentation in ovarian and liver cells of the juvenile channel catfish [96-98]. We observed an increase in apoptotic cells present inside and outside of the nervous system (Figure 3), suggesting activation of programmed cell death was not necessarily tissue specific. Systematic chemical analysis in zebrafish could help discern which components of the Macondo oil are responsible for induction of programmed cell death and whether induction is cell type specific.

### Selective impairment of neural crest cells is at the 'heart' of the problem

While increased cell death may play a role in the defects contributing to reductions in embryo size and shape, reductions in head size may be more directly associated with a lack of proper jaw formation. Defects in craniofacial development in response to crude oil or specific PAHs have been demonstrated previously [36]. The most prevalent and severe craniofacial defect we observed in response to Macondo crude oil WAF was the preferential loss of posterior pharyngeal cartilage elements (Figure 5A-F). As one might expect, the vasculature associated with these same pharyngeal arches was also reduced (Figure 4A-H). Most crude oils, or their components, cause cardiac edema, heart morphogenesis defects, and reduced circulatory function during embryogenesis of several fish species [9,36,38,47]. We hypothesized that the defects we observed in pharyngeal cartilage, vasculature development and heart morphogenesis were linked by an earlier disruption in the proper development of cranial neural crest cells, which are contributing precursors for all of these tissues.

Neural crest cells function as multipotent stem cells that actively delaminate from the dorsal neural tube and migrate along separate pathways across the entire anterior-posterior axis of an organism. During migration to their final destinations neural crest cells differentiate into a variety of cell types, some of which contribute to the development of pigment cells, peripheral nervous system, head cartilage, endothelial and smooth muscle vasculature, and portions of the heart [52-56]. Interestingly, recent investigations in amniotes have suggested pharyngeal arches and the heart are derived from the same "vagal" domain of neural crest cells lying at the intersection between the head and trunk axial positions (reviewed in [52]). This is of particular relevance since the most significant phenotypes we observed were restricted to the pharyngeal arch cartilage, arch vasculature and heart (Figure 4). Importantly, cardiac neural crest cells specifically migrate through the posterior pharyngeal arch pathways on route to the developing heart fields, where they differentiate into smooth muscle and endothelial cell derivatives that contribute to the morphogenesis of the outflow tract, septum, and valves of the heart [51,55].

We discovered that Macondo WAF-treated embryos show a specific reduction in one of the posterior arches, and a similar restricted reduction was seen in both *crestin *and *dlx2 *gene expression by cranial neural crest cells associated with the posterior pharyngeal arches (Figure 5G-P). Initially cranial neural crest cells migrate in three streams toward the presumptive arches, and the posterior-most stream undergoes a branching process to form three additional streams that establish the posterior most pharyngeal arches [99]. Therefore, we hypothesize that the causative toxins in the Macondo WAF may be affecting neural crest cell specification as it relates to their ability to carry out this branching step during posterior pharyngeal arch development. This spatially restricted defect in neural crest development is a remarkably targeted effect by crude oil, which suggests there are equally specific molecular pathways directly influenced by the components of the Macondo WAF; such as, the known neural crest regulators Wnt, Fibroblast growth factor, or the Bone Morphogenic Protein signaling pathway [51,100,101]. These pathways may be activated directly or independent of the *aryl hydrocarbon receptor 1 and 2*, which has been shown to be required for cardiac edema and heart morphogenesis defects in response to selective PAHs [37].

Despite the apparent lack of neural crest defects in the trunk of WAF-treated embryos, there was reduced circulation in the intersegmental blood vessels, and those vessels devoid of any circulation often showed excessive vascular branching (Figure 4L-O'). These elaborate and often forked branching patterns (Figure 4N', O', arrow) were reminiscent of an angiogenic process called intussusception, in which a vessel splits along its longitudinal axis and undergoes vascular remodeling [102,103]. While intussusception has not yet been described in zebrafish, others have demonstrated mouse retinal vasculature responds to hypoxic conditions by forming characteristic "vascular loops" [104]. In WAF-treated embryos vascular remodeling is seen only in intersegmental blood vessels that are devoid of circulation, which could represent an extreme but focused hypoxic event during which vascular looping might be observed (Figure 4N', bracket). We, therefore, hypothesize that the vascular remodeling observed in WAF-treated embryos is an indirect effect caused by reduced circulation in the intersegmental blood vessels due to a primary disruption in early neural crest-mediated heart development and function.

### Defects in patterning the peripheral nervous system impacts locomotor escape behaviors

Like most teleosts, zebrafish larvae evolved early stereotypical swimming patterns in response to touch stimuli that enable fast escape locomotor behaviors [105,106]. The inability to properly react to touch would have adverse consequences to larva survival. We observed that larvae exposed to the Deepwater Horizon crude oil had significantly reduced sensitivity to touch and disorganized swimming patterns relative to untreated controls (Figure 6). A previous study that examined the effects of specific PAHs on zebrafish locomotor behaviors did not detect any irregularities [36], suggesting that either the Macondo crude oil WAF possesses a unique component or a particularly toxic combination of known components impaired proper locomotor function. This could have unfortunate implications for the species of the Gulf of Mexico and Atlantic Ocean where even subtle reductions in larval or adult escape responses can be deadly. There is some precedent for this, as delayed escape behaviors have been documented in fiddler crabs in response to chronic exposure to No. 2 fuel oil polluting the sediment of Wild Harbor in Buzzards Bay, MA [107]. We sought to determine the developmental origin of the locomotor phenotype in WAF-treated embryos by assessing the anatomical organization of the neural circuitry and skeletal muscle necessary to respond to touch and yield functional swimming behaviors. Considering the complexity of the locomotor system, we were surprised to only detect specific deformations in the peripheral nervous and muscular systems; the neurons and astroglia within the central nervous system were normal in quantity and position (Figures 7, 8).

Similar to our findings of touch response defects, the peripheral sensory and motor axon defects and slow muscle patterning phenotypes, to the best of our knowledge, have never previously been documented. Specifically, Macondo crude oil WAF caused reduced sensory axonal branching along the entire trunk, and sporadic motor axon pathfinding errors directly associated with corresponding deformations in slow muscle fiber development. Reductions in sensory neuronal branches likely cause reductions in touch responses [108,109], which suggests that WAF-induced reductions in sensory axonal arbors contribute to the reduction in touch sensitivity. However, when a touch response was elicited in a WAF-treated larva, they exhibited disorganized swimming behaviors. While problems in sensory branching may contribute to these swimming errors, the cause is more likely a problem with stimulus transduction controlled by the downstream neural circuitry and muscular output [110].

WAF-treated embryos exhibit deformations in motor axon pathfinding and slow muscle development (Figure 8). These specific defects could definitely lead to impaired muscle contractions and swimming behaviors. Early muscle contractions have been shown to be required for the proper ventral trajectory of pathfinding sensory axons and their ability to exhibit appropriate self-avoidance behaviors to establish the mesh-like pattern of sensory branching [111]. However, this is not likely a significant influence as muscle and motor axon defects were not consistently seen throughout the trunk of WAF-treated embryos unlike the sensory axonal defects, nor were longitudinal pathfinding errors or significant axon to axon contact seen that are characteristically found following muscle contraction loss [111].

Motor axon pathfinding errors were only found in somites that exhibited corresponding slow muscle patterning defects, which strongly suggests motor axon pathfinding errors are not direct effects of the oil but rather indirect phenotypes in response to inappropriate guidance cues derived from earlier problems with somitogenesis and slow muscle patterning. This is in contrast to the sensory neuron branch reductions that are uniformly present throughout the trunk and, thus, likely a direct affect of exposure to the Macondo crude oil. Slow muscle cells have been shown to provide critical axon guidance cues that direct the proper pathfinding of motor axons [74,112]. This primary defect in early skeletal muscle development can be interpreted as two separable processes, in which there are changes to the highly stereotypical pattern of segmentation and then improper slow muscle positioning.

Within the musculature of the trunk we observed losses of somitic boundaries as well as the occurrence of inappropriate boundaries within the same somitic region (Figure 8B, C, F, G as examples). At this point we can only speculate how Macondo crude oil might be affecting somitogenesis. Segmentation in vertebrates is controlled by the precise coordination of a Notch-Delta mediated molecular clock that determines when a boundary will form and this process is paired with opposing anterior and posterior morphogenic gradients of Retinoic acid and Fibroblast growth factor that define the location of a segment boundary [77,79,113-116]. It is possible that some component within the Macondo crude oil might be impacting this somite molecular clock mechanism; however, it is also plausible that our WAF treatments were affecting the terminal step in boundary formation that involves the process of epithelialization and formation of the myotendinous junction rather than the timing of somitogenesis [117,118].

After a segment boundary has formed in the paraxial mesoderm of zebrafish, "adaxial" slow muscle precursor cells located adjacent to the notochord undergo substantial morphogenesis and movement to the outer-most edge of a somite to form a monolayer of elongated, slow-twitch muscle fibers [76,119,120]. While alterations in proper somite boundary formation would yield irregularly elongated slow muscle fibers, it has not been documented to cause alterations in the medial to lateral positioning of fibers, change the parallel positioning of the slow muscle array, nor cause early slow muscle loss [118]. Therefore, we hypothesize that in addition to somite formation defects, Macondo crude oil WAFs may be independently affecting some aspect of slow muscle specification and migration.

Hedgehog signaling is required for proper slow muscle cell specification and Cadherin cell adhesion molecules are required for proper slow muscle morphogenesis and movement, and their loss causes slow muscle positioning phenotypes similar to what we observed following WAF treatments [120-122] (Figure 8D, L, M as examples). While these conclusions related to muscle development are speculative, they provide a basis for a series of future experiments aimed at analyzing the effects of specific Macondo crude oil compounds on somitogenesis and muscle development, as well as examining the role of the Aryl hydrocarbon receptor signaling pathway in these processes [37].

Aside from these early somitogenesis and muscle fiber type patterning defects, a separate and later forming slow muscle degeneration phenotype was also variably present in some WAF-treated embryos. We found a number of embryos treated with the Macondo oil WAF had sporadic muscle fiber breaks and single sided detachment from the lamina, which subsequently exhibited cell death morphologies (Figure 8I, J). We interpret this phenotype as a specific, WAF-mediated muscle degeneration, as it is nearly identical to the muscle pathology seen in zebrafish genetic models of muscular dystrophy [80-85]. Importantly, this late muscle degeneration defect does not have corresponding motor axon pathfinding errors because it occurs after motor axons have already successfully reached their target cells. While these varied early and late muscle phenotypes have real consequences for embryonic health and locomotor function, the sporadic nature of these muscle phenotypes suggests they cannot fully account for the consistent errors in swimming behavior exhibited by WAF-treated embryos.

The abnormal locomotor behaviors exhibited by WAF-treated larvae could be due to defects in central nervous system function. Our level of analysis did not reveal any neuroanatomical defects; however, fine mapping of neural circuits or analysis of neuronal network activity could help elucidate specific cellular and molecular mechanisms disrupted by crude oil exposure. Moreover, it will be equally important to characterize the quality of myelin wrapping by oligodendrocytes and schwann cells, which contribute significantly to how well signals are conducted between neurons.

### Phenotypic changes over time

An unexpected finding of our research was the phenotypic reduction in certain defects over successive and identical experimental replicates. Specifically, apoptotic cell death and skeletal muscle phenotypes decreased in severity over the course of multiple experiments, while the phenotypes associated with neural crest development and sensory neuronal branching remained consistent. This observation suggests that different components within the Macondo crude oil might cause these phenotypes, and that some specific component(s) changed over the course of the use and storage of our Macondo crude oil sample. Importantly, we were able to reproduce the severity of both cell death induction and the severity of all the skeletal muscle phenotypes by exposing embryos to freshly mixed WAF preparations several times over the course of a single experiment. This confirms that Macondo crude oil is responsible for these phenotypes and, while the unidentified compound has reduced potency over time, it is still present and capable of impairing embryonic development. A recent study demonstrated that dissolved PAHs, rather than oil particles, are toxic to zebrafish embryos and cause edema, hemorrhaging, developmental delays, and abnormalities in cardiac function [38]. We propose a model in which the more readily dissolved PAHs are responsible for the neural crest derived phenotypes that lead to cardiac and craniofacial deformations, whereas the cell death and skeletal deformations may be caused by novel Macondo oil components, such as smaller ringed hydrocarbons that are more easily released from the WAF as a gas, or much heavier hydrocarbons that readily fall out of solution. Our findings will enable logical candidate approaches to analyze the role of individual components of Macondo oil in regulation of specific developmental processes during fish embryogenesis.

## Conclusions

These studies demonstrate that water-soluble components of Macondo crude oil cause specific teratogenic effects on developing zebrafish embryos. While exposure to Macondo oil WAFs did yield similar defects in cardiovascular and craniofacial development as with other crude oil types, we show this is likely due to an earlier impairment in the development of cranial neural crest cells. In addition, irregular locomotor escape responses in Macondo oil WAF treated larvae may be in part due to specific reductions in sensory axon branching as well as deformations in somite and slow muscle development. These results shed new light on a variety of specific developmental processes never before associated with crude oil exposure that provide a framework for future studies to define the molecular mechanisms governing crude oil teratogenesis. These novel phenotypes suggest that Macondo crude oil sampled during the Deepwater Horizon oil spill is toxic to zebrafish embryonic development, and, based on the high conservation of developmental mechanisms *Danio rerio *shares with other vertebrate species, the Macondo crude oil will similarly impact the embryonic development of native teleost species known to spawn in the Gulf of Mexico. We predict these findings can be used as a guide to initiate more efficient and systematic analyses of native species that may have been impacted by the original Deepwater Horizon oil spill and those species that could be affected by future leakage from the Macondo well.

## Methods

### Sampled Macondo crude oil

A one-liter sample of Macondo crude oil, MC 252 (Source Oil B; ID #: 20100617-121) from the Deepwater Horizon oil spill was provided by British Petroleum (BP) on September 8, 2010. This oil sample was collected from the riser insertion tube on the Enterprise by Entrix samplers during May 2-3, 2010. BP acknowledged that during sampling of this crude oil, Nalco EC9323A defoamer was being injected topside, while methanol with 10,000 ppm VX9831 oxygen scavenger/catalysts solution was being injected subsea. While the presence of these additional agents in the sample cannot be ruled out, it is unlikely they were incorporated into this crude oil sample due to the method of sampling directly through the riser tube. It was further acknowledged by BP that there was a variable amount of water in the oil samples collected in this manner. Material safety data sheets for the Source Oil B crude oil sample stated its major constituents were n-hexane, toluene, xylene, benzene, naphthalene, ethylbenzene, and hydrogen sulfide.

### Water accommodated fraction (WAF)

Following previously accepted mixing procedures, we created water accumulated fractions (WAF) of Macondo oil with autoclaved E3 embryo medium [40,123]. Specifically, the 1-liter container of Macondo crude oil was slowly inverted 20 times before collecting a sample to ensure consistent sampling over the course of the study. Oil dilutions of 1:10 in embryo medium were prepared in cap-sealed glass bottles that allowed for 20% air volume. Initial gas chromatography mass spectrometry (GCMS) analysis of WAF exposed to plastics detected n-butyl phthalate in solution, which is a plasticizer speculated to cause developmental effects on its own (data not shown, [124]). Therefore, only glass pipettes and glass petri dishes were used with WAF solutions. For initial characterization, WAF stock solutions were mixed with a magnetic stir bar in two different ways: (1) WAF stock was stirred at a slow, non-vortex inducing mixing speed [40]; and (2) WAF stock was stirred at a rate sufficient to produce a vortex equivalent to one-third the volume of the solution. Both solutions were mixed for 24 h, followed by a 2 h resting period before only the aqueous WAF phase was sampled. Unless otherwise noted, the embryos in the following experiments reported here were exposed to a 100% WAF solution derived from the 1:10 (crude oil to embryo media) vortex-mixed stock solution.

### WAF chemical analysis

To determine what the chemical composition was of the resulting WAF solutions made in embryo medium, Solid phase microextraction (SPME,100 mm polydimethylsiloxane) and gas chromatography mass spectrometry (GCMS, Agilent 7890A GC/5975C MSD) were performed. WAF samples were analyzed within one hour after the mixing procedure was completed. The SPME fiber was immersed for six minutes in a 15 mL vial of diluted WAF (25-fold dilution), with constant stirring, before insertion into the GC (helium carrier gas; splitless mode for first six minutes; 220°C inlet; oven at 35°C for four minutes, ramped 8°C/minute to 275°C, held at 275°C for three minutes). For consistency among WAF samples, standards for selected components (cyclopentane, methylcyclohexane, n-hexane, n-decane, n-tetradecane, benzene, toluene, xylenes, naphthalene, anthracene, phenanthrene, all from Supelco) were also made in diluted embryo medium (25-fold dilution). Benzene-d6 (Supelco) was added as an internal standard. An expanded explanation of the chemical analysis is provided in Additional files 1 and 2.

### Embryo collection and treatment

Fish lines were maintained in the ALAAC accredited Smith College Zebrafish Facility using standard husbandry techniques ([125]; Zebrafish International Resource Center). Fertilized eggs from wild type embryos (AB strain) and *Tg[fli:eGfp] *transgenic fish (TU/AB background, provided by the Lawson Lab, UMass-Medical School) were collected, washed in embryo medium, and incubated at 28.5°C. Unless otherwise stated, embryos were exposed to WAF solutions starting at 3.5 hours post-fertilization (hpf) and maintained in WAF solutions until a maximum of 5-days post fertilization (dpf). Control embryos were maintained in oil-free embryo medium and grown in a separate 28.5°C incubator to eliminate potential exposure to WAF gases. All treatments were carried out with either 20 mL of solution in 100 mm glass petri dishes or 10 mL of solution in 50 mm glass petri dishes. Prior to fixation or live embryo imaging, embryo chorions were removed by chemical digestion with Pronase (2 mg/ml, Sigma-Aldrich, St Louis, MO, USA) for three to five minutes.

### Behavioral analysis

In an effort to perform a more conservative assessment of locomotor behavior, analysis was carried out using a 50% WAF solution. Embryos exposed to either 50% or 100% WAF had similar locomotor responses (data not shown). To characterize larval swimming behavior at 48 hpf, a light touch stimulus was applied to the head of larvae. The minimum stimulus required to elicit a response was determined using pressure-specific Von Frey filaments. The touch-response was recorded using a high-speed camera (Fastec Imaging, San Diego, CA, USA) at 1,000 frames per second (fps). A 35 mm lens (Nikon, Melville, NY, USA) was used for magnification. To illustrate the responses, single frames taken at 20 mS intervals were overlaid in Adobe Photoshop (San Jose, CA, USA).

For kinematic analysis, the head-to-tail angles were calculated for each frame using automated software developed by the Downes Lab (Biology Department, University of Massachusetts, Amherst, MA, USA) [126]. In brief, pixel density was used to identify three landmarks along the larval body: the tip of the nose, the border between the yolk extension, and the tip of the tail. These three points form an angle, which was plotted over time using Microsoft Excel (Microsoft Corporation, Redmond, WA, USA). To calculate body bend frequency, a full body bend was defined as two intervals of more than 50 degrees of opposite directions. To determine the duration of a swimming episode, we measured the beginning of a swimming episode until the final time the body was straightened to within 20 degrees of being straight (defined as 0 degrees).

### Blood, circulation and vascular analysis

Visualization of blood in 3 dpf embryos was accomplished by staining hemoglobin with *o*-Dianisidine as previously described [127]. Blood circulation rates were determined by recording the amount of time a single blood cell took to travel through the dorsal aorta from somite 8 to somite 14. A total of three blood cells were recorded and their travel times averaged for each fish. Individual blood cells were easily visualized in live larvae at 3 dpf using an Olympus stereo microscope (Center Valley, PA, USA).

Microangiography was performed as previously published [49,128] and described in detail online [129]. Briefly, 3 dpf live control and WAF treated *tg[fli:eGfp] *larvae were anesthetized with Tricaine (MS222, Argent Chemical Laboratories, Inc, Redmond, WA, USA) to prevent skeletal muscle contractions, mounted ventral side up in 5% methylcellulose, and microinjected with Qtracker 655 non-targeted quantum dots (Invitrogen, Life technologies, Carlsbad, CA, USA) into the sinus venosus. Larvae were then laterally embedded in 0.75% agarose on glass bottom petri dishes (MatTek, Ashland, MA, USA), and imaged with an inverted Leica SP5 Laser Scanning Confocal Microscope (Lieca, Buffalo Grove, IL, USA) with resonant scanning. Live cell imaging of the fluorescently labeled green endothelial cells and red quantum dot-filled vasculature was captured at 20X using bi-directional scanning at 8,000 hz, 16-times line averaging at a 1024 × 1024 resolution, and with a 1.7 × digital zoom. Z-stacks with an optical slice thickness of 1 μm were collected of the entire trunk or portions of the head. Z-stack analysis and maximum intensity projections were performed using *Volocity *software (PerkinElmer, Waltham, MA, USA). Adobe Photoshop was used to align projections and reconstruct the entire trunk vasculature of individual larva as illustrated in Figure 4L-O.

### Immunohistochemistry and imaging

Embryos were fixed in 4% phosphate-buffered formaldehyde, except for anti-Islet1 labeling, which were fixed in 4% formaldehyde, 0.05% glutaraldehyde, 5 μM EGTA, 5 μM MgSO_4 _and 0.1% Triton-X-100. Primary antibodies used were mouse IgG_2b _anti-Acetylated Tubulin (AT, 1:800; Sigma); rabbit anti-Glial fibrillary acidic protein (Gfap, 1:500; Dako, Carpinteria, CA, USA), mouse IgG_1 _anti-Phospho-Histone H3 (PH3, 1:1000, Cell Signaling Technology, Inc., Danvers, MA USA), mouse IgG_1 _anti-slow muscle myosin (F59, 1:10; Developmental Studies Hybridoma Bank, University of Iowa, Department of Biology, Iowa City, Iowa, USA), rabbit anti-Gamma-aminobutyric acid (Gaba, 1:1000; Sigma), mouse IgG_1 _anti-3A10 (1:50, Developmental Studies Hybridoma Bank), and rabbit anti-activated Caspase 3 (1:500; BD Pharmigen, San Jose, CA, USA). All primary antibodies were diluted in blocking solution (see below). Secondary antibodies used were Alexa-594 goat anti-rabbit IgG, AlexaFluor-594 goat anti-mouse IgG_1_, AlexaFluor-647 goat anti-mouse IgG_2b_, and AlexaFluor-488 goat anti-mouse IgG_1_, all obtained from Invitrogen and diluted 1:200 in blocking solution. Immunocytochemistry was carried out as previously described [130] with minor modifications. Embryos were first dehydrated at -20°C for 20 minutes with 100% MeOH followed by 7 minutes with 100% Acetone. Further antibody penetration was achieved with protein digestion using Proteinase-K (10 μg/ml) for 2 minutes to 25 minutes depending on the age of the embryos. Embryos were incubated in blocking solution (0.5 g BSA; 25 ml Phosphate buffered saline, 0.1% Triton-X-100; 5% normal goat serum; 1% DMSO) prior to both primary and secondary antibody application.

Fluorescent, whole-mount images of fixed preparations were captured using the Zeiss AxioImager epifluorescent compound microscope with ApoTome for structural illumination. Embryos were dissected into separate trunk and head segments. Trunks were laterally mounted on a microscope slide, while heads for 3A10-labeled embryos were mounted dorsal side up. Imaging was performed with either 40× or 20× objectives and Z-stacks were collected with an averaging of 2 and 0.53 μm or 0.8 μm optical slice distance, respectively. An AxioCam Mrc (Carl Zeiss, Thornwood, NY, USA) was used to capture images on the AxioImager, and processing of Z-stacks and maximum intensity projections was done using Axiovision software (Carl Zeiss, Thornwood, NY, USA).

Whole-mount *in situ *hybridization at for *crestin *and *dlx2 *mRNA expression was completed as previously described [131]. Alcian Blue staining was performed to visualize cartilage development at 4 dpf and conducted as previously described [50]. *crestin, dlx2, tg(fli:Gfp) *labeled pharyngeal arches and Alcian Blue labeling were imaged on a Zeiss Lumar stereo microscope using an AxioCam HR and Axiovision software.

All figures were constructed using Adobe Photoshop. Any adjustments to image brightness, contrast or other properties were done to the entire image and identically performed between controls and WAF-treated data.

### Quantitation and statistics

Data collection was obtained in at least 20 embryos per 3 separate replicate experiments for all analyses. One exception was made for the microangiography live embryo imaging where labor intensive microinjection and live embryo mounting restricted analysis to 10 embryos per control and WAF-treated for each replicate. However, at least eight separate locations were imaged along the anterior to posterior axis of each embryo. Cell counts for PH3, Caspase, Gaba, Islet1, and Gfap were done on maximum intensity projections and quantified using the "counting tool" in Adobe Photoshop CS Extended version. Sensory neuron processes were quantified in Image J [132]. Briefly, 8-bit images were converted into binary images to calculate pixel density, and the average number of pixels for each group was analyzed for statistical significance. The results for each replicate were pooled and means calculated for most statistical analyses except where noted in the results. The two-sample T-Test was used to compare treatments with a stringency of *P *< 0.01 for all analyses, except when the data did not fit a normal distribution, in which case a non-parametric analysis (Kruskal-Wallis or Mann-Whitney tests) was performed. Statistical significance for *fli:Gfp *labeled arch formation and *dlx2 *expression was determined using a Fisher's Exact Test.

## Abbreviations

AT: Acetylated Tubulin; BP: British Petroleum; dpf: days post-fertilization; fps: frames per second; Gaba: Gamma-aminobutyric acid; GCMS: gas chromatography mass spectrometry; Gfap: Glial fibrillary acidic protein; hpf: hours post-fertilization; NOAA: National Oceanic and Atmospheric Administration; PAH: polycyclic aromatic hydrocarbons; PH3: Phosphorylated Histone H3; SPME: solid phase microextraction; WAF: water accumulated fraction.

## Competing interests

The authors declare that they have no competing interests.

## Authors' contributions

TS participated in experiment design and played a role in carrying out many of the experiments in this study with particular efforts on all cardiovascular, craniofacial, and neural crest analysis. AU contributed significantly to initial experimental design of this study as well as carried out most of the initial replicates. TF carried out the behavioral analysis of touch responsiveness and the kinematics of swimming. DP carried out a majority of the analysis of WAF chemical composition. SC carried out a majority of the refreshing WAF treatments and experiments as well as performed many of the statistical analyses. DO assisted in a majority of embryo treatments as well as supported immunocytochemistry experiments. RB provided assistance on all immunocytochemistry, embryo treatments and imaging on the AxioImager. GD designed, monitored and participated in the behavioral analysis. SH designed, monitored and participated in the chemical analysis of WAF components. RS assisted in data collection for the analysis of cell death. MCL performed the initial experiments on slow muscle development. KH designed and supported the statistical analysis of collected data. LK supported many of the final experiments of this work contributing directly to the acquisition of *fli:GFP *and *dlx2 *data. MB conceived of the study, substantially contributed to the design, coordination, and implementation of many of the experiments, with particular contributions to microangiography procedures, and cartilage and neural crest imaging. MB participated in all data and statistical analysis and drafted the manuscript. All authors read, contributed feedback to, and approved the final manuscript.

## Authors' information

MB, the corresponding author, is a neurodevelopmental biologist serving as an assistant professor in the Biological Sciences department and member of the Neuroscience program at Smith College.

## Supplementary Material

Additional file 1 **Initial assessment of water accumulated fractions**. This file provides greater explanation of the methods and results associated with our WAF mixing procedure, as well as initial assessments of the effects of different WAF concentrations on the development of zebrafish gross morphology. Click here for file

Additional file 2 **Expanded WAF chemical analysis**. This file provides a detailed list of all the compounds and concentrations discovered following gas chromatography mass spectrometry analysis of our WAF. *Supplemental Table S1*: GCMS parameters for Agilent 7890A GC/5975C MSD. *Supplemental Table S2: *Aqueous solubilities and Henry's constants for selected hydrocarbons. *Supplemental Table S3: *Most prominent GCMS peaks in WAF. *Supplemental Figure S1: *Relative detection efficiencies of the SPME/GCMS method for selected aromatics and alkanes. *Supplemental Figure S2: *Total ion chromatogram. *Supplemental Figure S3: *Total ion chromatograms for the aqueous portion of a WAF sample and for a sample that includes some of the oily layer. Click here for file
