# Supplementary material for: Macondo crude oil from the Deepwater Horizon oil spill disrupts specific developmental processes during zebrafish embryogenesis
Source: BMC Biol. 2012 May 4;10:40. doi: 10.1186/1741-7007-10-40 (PMC3364156; doi:10.1186/1741-7007-10-40)
Supplement: Additional file 1 — Initial assessment of water accumulated fractions. This file provides greater explanation of the methods and results associated with our WAF mixing procedure, as well as initial assessments of the effects of different WAF concentrations on the development of zebrafish gross morphology. [file 1741-7007-10-40-S1.PDF]

Additional File 1. Initial assessment of water accumulated fractions.

Crude oil directly sampled from the lower riser of the Deepwater Horizon blow out between the dates of May 2 and May 3 of 2010 was provided by British Petroleum (BP). Water accumulated fractions (WAF) of the Macondo crude oil were made in embryo medium at a 1:10 dilution. Initial characterization of the WAF stock solution included analysis of two mixing procedures, one with and one without vortex-induced stirring in accordance with conventional methods [16]. Furthermore, a series dilution of the WAF stock solution was created and applied to wild type zebrafish embryos from 3.5 hpf until 5 dpf. For this initial analysis only gross morphological phenotypes and mortality were assayed.

Stirring the oil-embryo medium mixture with a vortex equivalent to 30% the volume of the solution produced WAF solutions causing more severe and consistent gross morphological phenotypes as compared to non-vortex mixed preparations (data not shown). Serial dilutions of mixed WAF stock solution produced a dose response in both the severity and frequency of the gross phenotypes, such that dilutions with 10% or less of the mixed WAF did not show any observable gross phenotypes, significant mortality, or locomotor behavioral defects. Using 25% of the mixed WAF did show variable presence of embryos with some gross morphological phenotypes such as cardiac edema and dorsal tail curvature, while exposure to a 50% WAF dilution exhibited increases in all of these characters near 100% WAF levels. Averaging over 7 separate replicates, embryos treated in 50% or 100% of the mixed WAF had 30% mortality by 5 dpf. Survival beyond 5 dpf requires the ability to aggressively feed and swim, traits that the gross morphological deformations and reduced swim bladder would not permit.

To maximize the opportunity to observe even subtle phenotypes associated with exposure to Macondo crude oil, we conducted all embryo treatments at the 100% concentration of the vortex-mixed, 1:10 WAF stock solution. Only during our analysis of locomotor behavior was 50% WAF solution used.
