# Supplementary material for: Macondo crude oil from the Deepwater Horizon oil spill disrupts specific developmental processes during zebrafish embryogenesis
Source: BMC Biol. 2012 May 4;10:40. doi: 10.1186/1741-7007-10-40 (PMC3364156; doi:10.1186/1741-7007-10-40)
Supplement: Additional file 2 — Expanded WAF chemical analysis. This file provides a detailed list of all the compounds and concentrations discovered following gas chromatography mass spectrometry analysis of our WAF. Supplemental Table S1: GCMS parameters for Agilent 7890A GC/5975C MSD. Supplemental Table S2: Aqueous solubilities and Henry's constants for selected hydrocarbons. Supplemental Table S3: Most prominent GCMS peaks in WAF. Supplemental Figure S1: Relative detection efficiencies of the SPME/GCMS method for selected aromatics and alkanes. Supplemental Figure S2: Total ion chromatogram. Supplemental Figure S3: Total ion chromatograms for the aqueous portion of a WAF sample and for a sample that includes some of the oily layer. [file 1741-7007-10-40-S2.PDF]

Additional file 2. Expanded WAF chemical Analysis

Samples for gas chromatography mass spectrometry (GCMS) analysis were further diluted 1:25 of WAF in water. To maintain the same concentrations of salts throughout, standards were made in embryo medium diluted similarly (1:25). For each sample, the SPME fiber (100mm polydimethylsiloxane) was exposed to 15.00 mL of liquid in a 15 mL PTFE-capped vial for 6 minutes at room temperature, with stirring, prior to injection into the GCMS (Agilent 7890A GC / 5975C MSD). GCMS parameters are in Sup. Table 1.

Our method of chemical analysis had a relatively high sensitivity to higher-mass aromatics, and less sensitivity to alkanes, with differences spanning almost 2 orders of magnitude. For the same concentration (4-400 ppb standards), peak areas increased for aromatics with increasing mass, to the point that the detection efficiency of naphthalene was over 10 times that of benzene (Sup. Fig. 1). In contrast, no mass-dependent pattern was discernable for alkanes, but for the same concentration, all alkanes had peaks smaller than benzene. Detection efficiency for some alkanes was almost ten times lower than for benzene (Sup. Fig. 1). We estimate our detection limit for cyclopentane and hexane to be on the order of 25-30 ppb. Given the 1:25 dilution, scaled, our detection limits for these low-mass alkanes translates to approximately 600-700 ppm in the WAF.

A representative total ion chromatogram for the diluted WAF is shown in Sup. Fig. 2; Sup. Table 3 lists the corresponding individual components. Aromatics (blue squares) were most prominent (Sup. Fig. 2). While less prominent in the total ion chromatogram, due lower sensitivity, alkanes (red circles) were present in similar concentrations (Sup. Table 4).

Our chemical analysis was reproducible with a standard deviation of 7% for 6 repeated analyses of the same naphthalene concentration (100 ppb). Alkane standards, however, showed less reproducibility. We attribute this to their lower solubility and higher volatilities from water (Sup. Table 2), as well as their lower detection efficiencies. As a result, standard curves for alkanes contain significantly greater uncertainty, and the systematic error in alkane concentrations could be as high as a factor of 2.

The lower solubility and higher volatilities of alkanes from water also account for high variability in alkane measurements from the WAF (Sup. Table 4). Inclusion of any oily layer in the sample for analysis, for example by shaking the solution to create an emulsion with the aqueous phase, added more of the less water-soluble compounds (Sup. Fig. 3).

Supplemental Table 1. GCMS parameters for Agilent 7890A GC / 5975C MSD.

Inlet temperature	220°C
Oven temperature	35°C for 4 minutes, 8°C per minute increase up to 275°C, 275 °C for 3 minutes
Mode	Splitless for first 6 minutes
Flow rate	105 mL/minute splitless<br>100 mL/minute split
Mass range	45-400

Supplemental Table 2. Aqueous solubilities and Henry's constants for selected hydrocarbons, taken from the *CRC Handbook of Chemistry and Physics*.

		Solubility (ppm)	Henry's constant (kPa m^3 mol^{-1})
▲	Cyclopentane	157	19.1
▲	Methylcyclohexane	51	43.3
●	Hexane	11	183
●	Decane	0.015	479
●	Dodecane	0.0037	750
●	Tetradecane	0.12	-
▣	Benzene	1780	0.557
▣	Toluene	5310	0.660
▣	Ethylbenzene	161	0.843
▣	o- and m- Xylene	171, 161	0.551, 0.730
▣	p-Xylene	181	0.690
▣	Naphthalene	31.6	0.043
▣	Methylnaphthalene	28.1	0.045, 0.051

Supplemental Table 3. Most prominent GCMS peaks in WAF, with assignments by matches to NIST Library mass spectra. In cases with more than one assignment with a similar match, the top 2 or 3 are listed.

		Retention time (minutes)	Match to NIST library (%)	% Total area
▣	Toluene	4.035	95	26.2%
▣	o, m-Xylene	6.926	95	12.8%
▣	Benzene	2.158	91	11.1%
▣	p-Xylene	7.551	95	6.4%
▣	Benzene, 1,2,3-trimethyl- / 1,2,4-trimethyl-	10.057	97	3.6%
▣	Naphthalene	14.017	91	3.5%
▣	Naphthalene, 1-methyl- / 2-methyl-	16.035	95	3.2%
▣	Ethylbenzene	6.715	91	2.5%
▣	Naphthalene, 2-methyl- / 1-methyl-	16.33	94	2.1%
▣	Benzene, 1-ethyl-2-methyl- / 1-ethyl-3-methyl- / 1-ethyl-4-methyl-	9.32	95	2.0%
▣	Benzene, 1,3,5-trimethyl- / 1,2,3-trimethyl-	10.709	95	1.7%

◘	Naphthalene, 1,7-dimethyl- / 2,3-dimethyl-	18.13	96	1.2%
◘	Naphthalene, 2,3-dimethyl- / 2,7-dimethyl- / 1,6-dimethyl-	17.887	97	1.1%
◘	Benzene, 1,2,3-trimethyl- / 1,2,4-trimethyl- / 1,3,5-trimethyl-	9.478	97	1.0%
●	Pentadecane	19.336	95	0.9%
◘	Benzene, 1-ethyl-2-methyl-	9.743	95	0.8%
◘	Naphthalene, 2,3-dimethyl- / 1,6-dimethyl-	18.183	97	0.8%
●	Hexadecane	20.816	96	0.6%
●	Tetradecane	17.772	96	0.6%
●	Heptadecane	22.221	98	0.5%
●	Octadecane	23.554	97	0.4%
●	Nonadecane, 9-methyl-	24.826	80	0.4%
●	Hexadecane, 2,6,11,15-tetramethyl- / 2,6,10,14-tetramethyl-	22.302	83	0.4%
◘	Benzene, 1-methyl-2-(1-methylethyl)- / 2-ethyl-1,3-dimethyl-	12.083	58	0.4%
◘	Naphthalene, 1,4-dimethyl- / 2,7-dimethyl- / 1,5-dimethyl-	18.444	95	0.4%
◘	Naphthalene, 1,3-dimethyl-	18.5	87	0.4%
◘	Naphthalene, 2,3,6-trimethyl- / 1,6,7-trimethyl-	20.107	98	0.4%
●	Tridecane	16.106	94	0.3%
◘	Benzene, propyl-	9.121	80	0.3%
◘	Benzene, 1-ethyl-3,5-dimethyl- / 2-ethyl-1,3-dimethyl-	13.417	94	0.3%
◘	Naphthalene, 1,6,7-trimethyl- / 1,4,5-trimethyl-	19.858	97	0.3%
◘	Naphthalene, 2-ethenyl- / Biphenyl	17.474	90	0.3%
◘	Naphthalene, 1,5-dimethyl- / 1,3-dimethyl- / 1,6-dimethyl-	18.696	94	0.3%
●	Tetratetracontane	26.038	90	0.3%
◘	Naphthalene, 1-ethyl-	17.713	96	0.3%
◘	Phenanthrene	23.374	91	0.3%
◘	Naphthalene, 1,6,7-trimethyl- / 2,3,6-trimethyl-	20.141	96	0.3%
◘	1,1'-Biphenyl, 3-methyl- / 4-methyl-	19.162	93	0.3%
◘	Benzene, 1,2,3,5-tetramethyl- / 2-ethyl-1,3-dimethyl- /1-ethyl-2,4-dimethyl-	11.944	91	0.3%
◘	Benzene, 1-methyl-3-propyl- / 1-methyl-2-propyl-	11.365	94	0.2%
◘	Benzene, 1,2,4,5-tetramethyl- / 1-ethyl-2,4-dimethyl-	12.786	91	0.2%
●	Dodecane, 2,6,10-trimethyl- / Hexacosane	23.679	78	0.2%
●	Pentadecane, 2,6,10-trimethyl-	21.515	72	0.2%
◘	Benzene, (1-methylethyl)-	8.4	91	0.2%
◘	Naphthalene, 1,6,7-trimethyl- / 2,3,6-trimethyl-	20.352	95	0.2%
◘	Naphthalene, 1,6,7-trimethyl-	19.787	97	0.2%
◘	Naphthalene, 1,4,6-trimethyl- / 1,6,7-trimethyl-	19.457	94	0.2%
◘	Benzene, 1-ethyl-2,4-dimethyl- / 1-ethyl-3,5-dimethyl-	11.521	94	0.2%

□	Fluorene	20.635	83	0.2%
□	9H-Fluorene, 9-methyl- / 3-methyl- / 1-methyl-	22.373	90	0.2%
●	Undecane, 2,3-dimethyl- / Dodecane, 2,6,10-trimethyl-	18.755	78	0.2%
□	Benzene, 1-ethyl-2,3-dimethyl- / 2-ethyl-1,4-dimethyl-	12.509	91	0.2%
●	Hexatriacontane / Heptacosane / Tetratetracontane	27.198	86	0.2%
□	Benzene, (2-methyl-2-propenyl)- / (1-methyl-1-propenyl)- / 1H-Indene, 2,3-dihydro-4-methyl-	13.37	86	0.2%
□	Benzene, 1,2,3,5-tetramethyl- / 1-ethyl-2,3-dimethyl- / 1,2,4,5-tetramethyl-	12.699	80	0.2%
▲	Cyclohexane, undecyl- / (2-methylpropyl)-	21.63	37	0.2%
●	Dodecane	14.322	93	0.2%
□	1,4-Methanonaphthalene,1,4-dihydro-9-((1-methylethylidene)- / 1,1'-Biphenyl, 3,4'-dimethyl- / 3,3'-Dimethylbiphenyl	20.887	55	0.2%
□	Naphthalene, 1,2,3,4-tetrahydro-	13.594	60	0.2%
□	Benzene, 2-ethyl-1,4-dimethyl- / 1-ethyl-2,3-dimethyl- / 4-ethyl-1,2-dimethyl-	11.912	91	0.2%
□	Benzene, (3-methyl-2-butenyl)-/ a,b,b-Trimethylstyrene	14.3	76	0.2%
□	Benzene, 1-methyl-2-propyl- / 1-methyl-3-propyl-	11.443	64	0.2%
□	Indane	10.989	83	0.2%
□	Benzene, 1-methyl-2-propyl- / Benzene, 1-methyl-4-propyl-	11.692	90	0.2%
□	Naphthalene, 1,4,6-trimethyl- / 2,3,6-trimethyl-	20.713	93	0.1%
□	Phenanthrene, 3-methyl- / Anthracene, 9-methyl- / Anthracene, 2-methyl-	24.919	72	0.1%
□	Naphthalene, 1,4,6-trimethyl- / 1,4,5-trimethyl-	19.544	50	0.1%
●	Tetracontane, 3,5,24-trimethyl- / 2,6-Dimethyldecane	28.311	50	0.1%
□	1H-Indene, 2-phenyl- / Anthracene, 2-methyl- / 1H-Cyclopropa[l]phenanthrene,1a,9b-dihydro-	25.155	72	0.1%
□	Azulene, 4,6,8-trimethyl-	20.387	64	0.1%

Supplemental Table 4. Concentrations (ppm) for selected components in 4 WAF samples. Samples in grey had visible oily layers that were mixed in before analysis.

		5/23/11	5/31/11	6/20/11	6/20/11	6/27/11
▲	Cyclopentane	0.78	<0.64	1.40	0.69	0.68
▲	Methylcyclohexane	1.28	0.90	1.16	0.91	0.83
●	Hexane	0.87	<0.75	1.14	0.77	0.75
●	Decane	7.84	2.27	0.07	2.35	0.01
●	Dodecane*	43**	8.27	0.39	12.62	0.63
●	Tetradecane	49**	8.91	0.31	10.64	1.60
●	Hexadecane*	66**	6.91	0.26	8.16	1.68
●	Octadecane*	42**	5.61	0.25	6.62	1.23
□	Benzene	7.32	4.24	12**	8.23	8.58

□	Toluene	6.58	3.19	7.87	5.92	5.92
□	Ethylbenzene	0.70	0.34	0.53	0.47	0.43
□	o- and m- Xylene	3.05	1.22	2.08	1.80	1.62
□	p-Xylene	1.40	0.64	0.95	0.86	0.84
□	Naphthalene	0.78	0.55	0.46	0.59	0.49
□	Methylnapthalene***	0.60	0.24	0.18	0.38	0.24

* Alkane concentrations marked with an asterisk are estimated, assuming similar detection efficiencies with tetradecane.
** Integrations fell outside the range over which standard curves were collected.
***Methylnapthalene concentrations are for both 1- and 2-methylnapthalene combined, and are based on the extrapolated mass-dependence of detection efficiencies for aromatics (Figure 1).

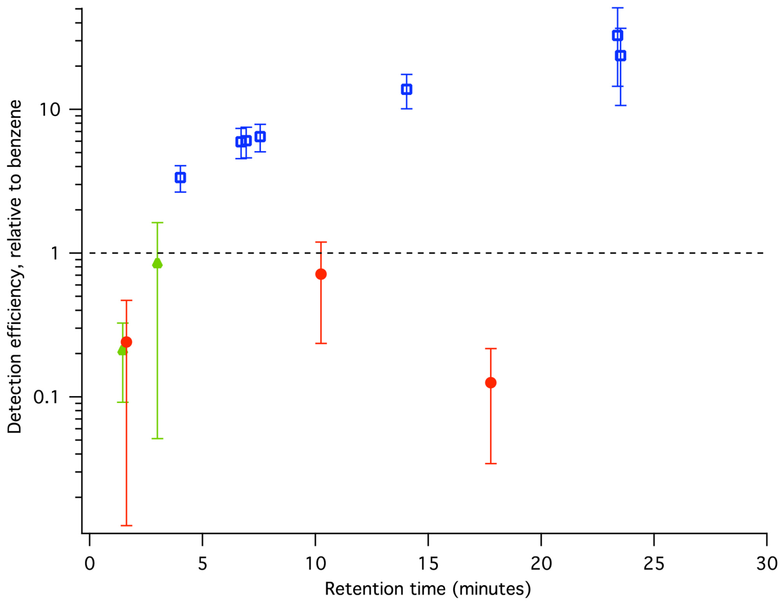

Supplemental Figure 1. Relative detection efficiencies the SPME/GCMS method for selected aromatics (blue squares), cyclic alkanes (green triangles) and long-chain alkanes (red circles). Our method was more sensitive to larger aromatics than to benzene. In order of increasing retention time the blue squares in the upper half of the graph represent toluene, o- and m-xylene, p-xylene, naphthalene, anthracene, and phenanthrene. Our method was less sensitive to alkanes. In order of increasing retention time, the data points in the lower half of the graph represent cyclopentane (green), n-hexane (red), methylcyclopentane (green), n-decane (red), and n-tetradecane (red).

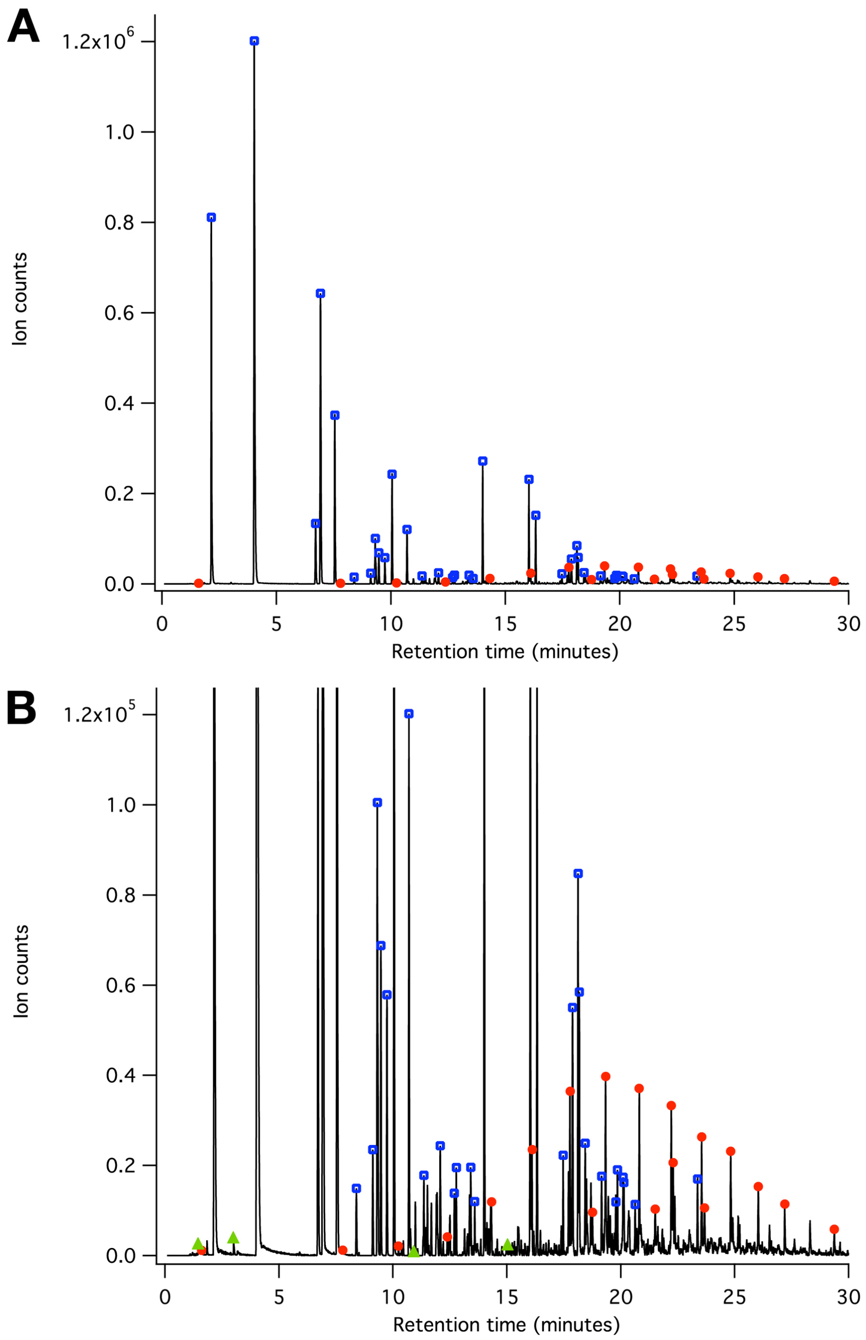

Supplemental Figure 2. (A) Total ion chromatogram for a representative WAF sample (1:25 dilution). (B) 10X enlargement of (A) to show the lower-intensity alkanes (red circles). Blue squares mark selected compounds identified by matching to the NIST Library as aromatics (mostly alkylated benzenes and naphthalenes). Red circles mark selected compounds identified as alkanes; green triangles indicate cyclic alkanes.

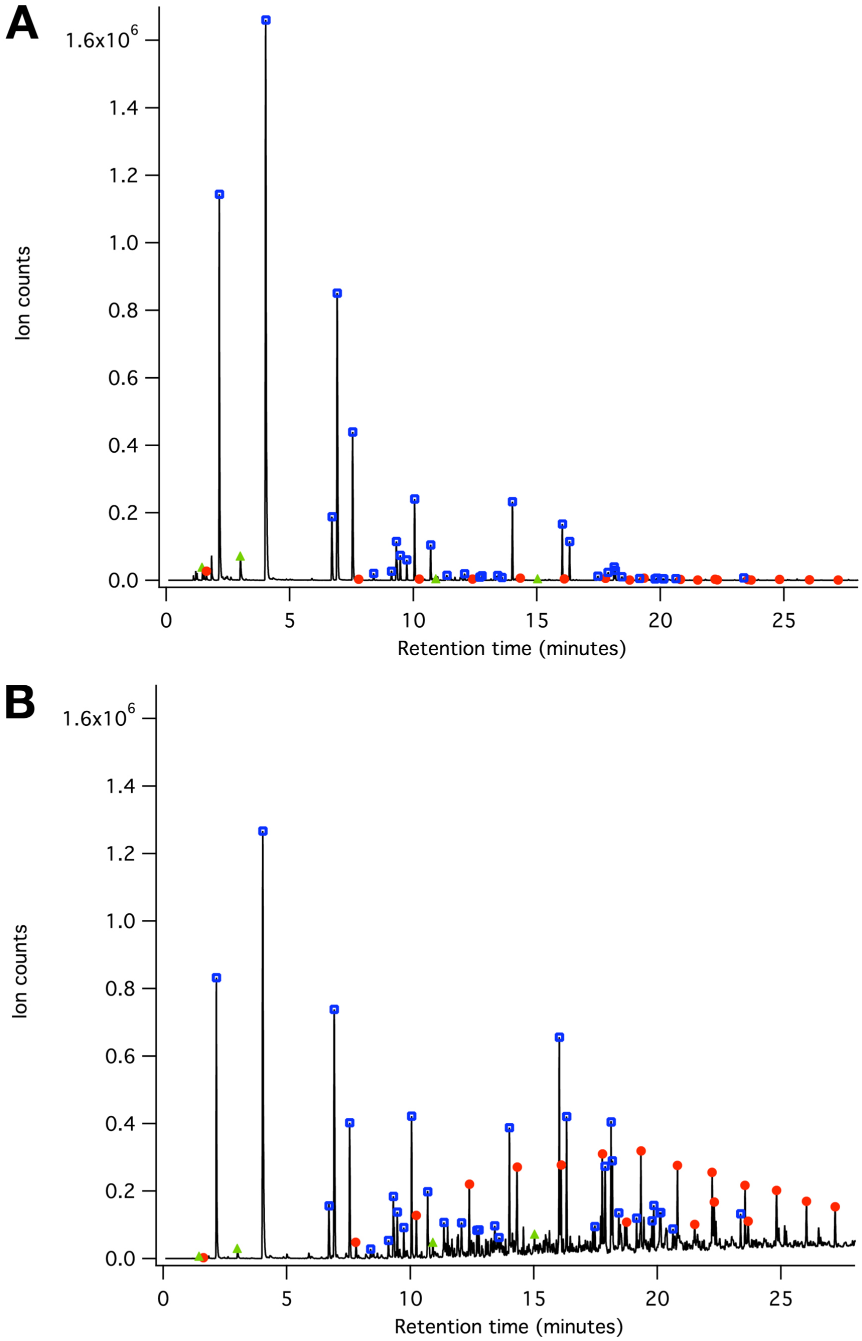

Supplemental Figure 3. Total ion chromatograms for the aqueous portion of a WAF sample (A), and for a sample that includes some of the oily layer (B). Blue squares mark selected compounds identified by matching to the NIST Library as aromatics (mostly alkylated benzenes and naphthalenes). Red circles mark selected compounds identified as alkanes; green triangles indicate cyclic alkanes.
